# Quantum state design and emergent confinement mechanism in measured tensor network states

**DOI:** 10.1038/s41534-026-01298-9

**Published:** 2026-07-04

**Authors:** Guglielmo Lami, Andrea De Luca, Xhek Turkeshi, Jacopo De Nardis

**Affiliations:** 1 Laboratoire de Physique Théorique et Modélisation, CNRS UMR 8089, CY Cergy Paris Université, Cergy-Pontoise, Cedex France; 2 JEIP, UAR 3573 CNRS, Collége de France, PSL Research University, 11 Place Marcelin Berthelot, Paris, Cedex France; 3 Laboratoire de Physique de l’Ecole Normale Superieure, ENS, Universite PSL, CNRS, Sorbonne Universite, Universite Paris-Cité, Paris, France; 4 Institut für Theoretische Physik, Universität zu Köln, Köln, Germany

**Keywords:** Mathematics and computing, Physics

## Abstract

Randomness is a fundamental aspect of quantum mechanics, arising from the measurement process that collapses superpositions into definite outcomes according to Born’s rule. Generating large-scale random quantum states is crucial for quantum computing and many-body physics, yet it remains a key challenge. We present a practical method based on local measurements of random Tensor Networks, focusing on random Matrix Product States (MPS) generated by two distinct quantum circuit architectures, both feasible on near-term devices. We certify the emergent quantum randomness using the frame potential and establish a mapping between its behavior and the statistical mechanics of a domain wall particle model. In both architectures, the effect of quantum measurements induces a nontrivial confinement mechanism, where domain walls are either trapped by an external potential or bound in pairs to form meson-like excitations. Our results, supported by both exact analytical calculations and numerical simulations, suggest that confinement is a general mechanism underlying random state generation in broader settings with local measurements, including quantum circuits and chaotic dynamics.

## Introduction

Randomness is a fundamental feature of quantum mechanics, manifested through measurement-induced collapse of quantum superpositions with probabilities dictated by the wave function amplitudes. Beyond its foundational significance, quantum randomness plays a crucial role in quantum technologies^1–3^, from cryptographic protocols to benchmarking quantum devices^4,5^. Yet, generating and characterizing large-scale random quantum states remains a fundamental challenge.

A central notion in this context is that of quantum state designs^6–8^, which quantify how well an ensemble of states approximates the uniform probability measure on the Hilbert space (usually dubbed the Haar measure). Although random quantum circuits have been extensively studied as a means to achieve such designs^9–11^, the question of how randomness emerges in more constrained quantum architectures remains open. In particular, Tensor Network states^12,13^–ubiquitous in condensed matter physics^14,15^, quantum simulations^16,17^, and holography^18–20^– offer a natural yet unexplored framework to investigate this question.

In this work, we study the emergence of quantum randomness in random tensor networks subjected to local measurements^21^. Specifically, we consider the ensemble of states obtained by projecting a random matrix product state (RMPS) or a random projected entangled pair state (PEPS) onto a measurement basis. The statistical properties of the resulting projected ensemble are analyzed using the frame potential, a standard tool for quantifying proximity to quantum designs. Through a mapping to a statistical mechanics model of interacting domain walls in replica space, we uncover a general mechanism: randomness emerges from a confinement effect. Here, confinement refers to an emergent potential that favors domain wall localization near subsystem boundaries or binding into meson-like pairs. This phenomenon echoes the confinement in quantum chromodynamics (QCD)^22,23^ and condensed matter systems^24–26^, where quarks are bound by a linearly growing potential and never observed in isolation. In our setting, the combination of quantum measurements and the circuit’s geometry binds the domain wall excitations, enhancing the uniformity of the ensemble, yet producing clear deviations from the Porter-Thomas distribution, the latter being expected only for fully random states^27^. Notice that, although the confinement of excitations takes place within the replica space, it has observable effects on the randomness of the ensemble. Indeed, the mechanism we identify directly influences the frame potential of the projected ensemble, which in turn provides a quantitative measure of the difference in the average value of any observable between the projected and Haar ensembles. Going beyond the realm of tensor network states, our findings suggest that such confinement is a generic mechanism for the emergence of quantum randomness, with implications for random quantum circuits, chaotic quantum dynamics, and entanglement structure in many-body systems. By elucidating this fundamental mechanism, our work provides a new perspective on the structure of quantum ensembles and the generation of randomness in quantum matter.

## Results

### Quantum state design via measurements

Nontrivial forms of quantum randomness emerge when multiple constituencies (e.g., qudits) interact via random quantum operations—as in deep circuits with randomly chosen gates. In an *N*-qudit quantum system described by a *D* = *d*^*N*^-dimensional Hilbert space ${\mathcal{H}}$, random (pure) states ${\mathcal{H}}$ can be generated by initializing the system in the product state ${\left|0\right\rangle }^{\otimes N}$ and applying a global unitary *U* sampled from the Haar measure over the unitary group ${\mathcal{U}}(D)$. The resulting ensemble, $\mathrm{Haar}=\{\left|\psi \right\rangle =U{\left|0\right\rangle }^{\otimes N},\,U \sim {\mathcal{U}}(D)\}$, is maximally random but infeasible for moderate *N*.

Hence, one seeks efficient ensembles ${\mathcal{E}}$ of pure states and quantifies randomness via the *k*-th frame potential, ${{\mathcal{F}}}_{{\mathcal{E}}}^{(k)}={{\mathbb{E}}}_{\psi ,\psi {\prime} \sim {\mathcal{E}}}[| \langle \psi | {\psi }^{{\prime} }\rangle {| }^{2k}]$, compared to the frame potential of the Haar ensemble, which (for large Hilbert space dimension *D*) is ${{\mathcal{F}}}_{{\rm{Haar}}}^{(k)}\simeq {D}^{-k}k!$. When ${{\mathcal{F}}}_{{\mathcal{E}}}^{(k)}={{\mathcal{F}}}_{{\rm{Haar}}}^{(k)}$, the ensemble ${\mathcal{E}}$ becomes *statistically indistinguishable from* Haar *for any observable acting on at most k identical* *copies of the system*; such an ensemble is denoted as a *k*-design^28,29^.

A resource-efficient framework to generate a *k*−design uses projective measurements^21,30–37^. The system is split into *A* (*N*_*A*_ qudits, dimension *d*_*A*_) and *B* (*N*_*B*_ qudits, *d*_*B*_). Measuring *B* on copies of $\left|\psi \right\rangle$ yields the projected ensemble

1 $${\mathcal{P}}[\left|\psi \right\rangle ]=\{\left|\psi ({{\boldsymbol{z}}}_{B})\right\rangle ,\,\,p({{\boldsymbol{z}}}_{B})\},$$

with ${{\boldsymbol{z}}}_{B}=({z}_{1},\ldots ,{z}_{{N}_{B}})$, measurement probabilities $p({{\boldsymbol{z}}}_{B})={\rm{Tr}}[({{\mathbb{1}}}_{A}\otimes \left|{{\boldsymbol{z}}}_{B}\right\rangle \left\langle {{\boldsymbol{z}}}_{B}\right|)\left|\psi \right\rangle \left\langle \psi \right|]$, and $\left|\psi ({{\boldsymbol{z}}}_{B})\right\rangle =({{\mathbb{1}}}_{A}\otimes \left\langle {{\boldsymbol{z}}}_{B}\right|)\left|\psi \right\rangle /\sqrt{p({{\boldsymbol{z}}}_{B})}$. Although its first moment reproduces the conventional reduced density matrix, ${\rho }_{{\mathcal{P}}[\left|\psi \right\rangle ]}={{\mathbb{E}}}_{\phi \sim {\mathcal{P}}[\left|\psi \right\rangle ]}[\left|\phi \right\rangle \left\langle \phi \right|]={{\rm{Tr}}}_{B}[\left|\psi \right\rangle \left\langle \psi \right|]$, the projected ensemble encodes structure beyond this first moment. The information is encoded in the statistical fluctuations of the ensemble, and can be captured by the frame potential^38,39^

2 $$\begin{array}{l}{{\mathcal{F}}}_{{\mathcal{P}}[\left|\psi \right\rangle ]}^{(k)}\,=\,\mathop{\sum }\limits_{{{\boldsymbol{z}}}_{B},{{\boldsymbol{z}}}_{B}^{{\prime} }}p({{\boldsymbol{z}}}_{B})p({{\boldsymbol{z}}}_{{\bf{B}}}^{{\boldsymbol{{\prime} }}})| \langle \psi ({{\boldsymbol{z}}}_{B})| \psi ({{\boldsymbol{z}}}_{B}^{{\prime} })\rangle {| }^{2k}=\\ \,\,\,\,\,\,\,\,\,\,\,\,\,=\,\mathop{\sum }\limits_{{{\boldsymbol{z}}}_{B},{{\boldsymbol{z}}}_{B}^{{\prime} }}p{({{\boldsymbol{z}}}_{B})}^{1-k}p{({{\boldsymbol{z}}}_{B}^{{\prime} })}^{1-k}| \langle \widetilde{\psi }({{\boldsymbol{z}}}_{B})| \widetilde{\psi }({{\boldsymbol{z}}}_{{\boldsymbol{B}}}^{{\boldsymbol{{\prime} }}})\rangle {| }^{2k},\\ \end{array}\,$$

where $\left|\widetilde{\psi }({{\boldsymbol{z}}}_{B})\right\rangle =({{\mathbb{1}}}_{A}\otimes \left\langle {{\boldsymbol{z}}}_{B}\right|)\,\left|\psi \right\rangle$ are the unnormalized post-measurement states in *A*.

**Fig. 1:**
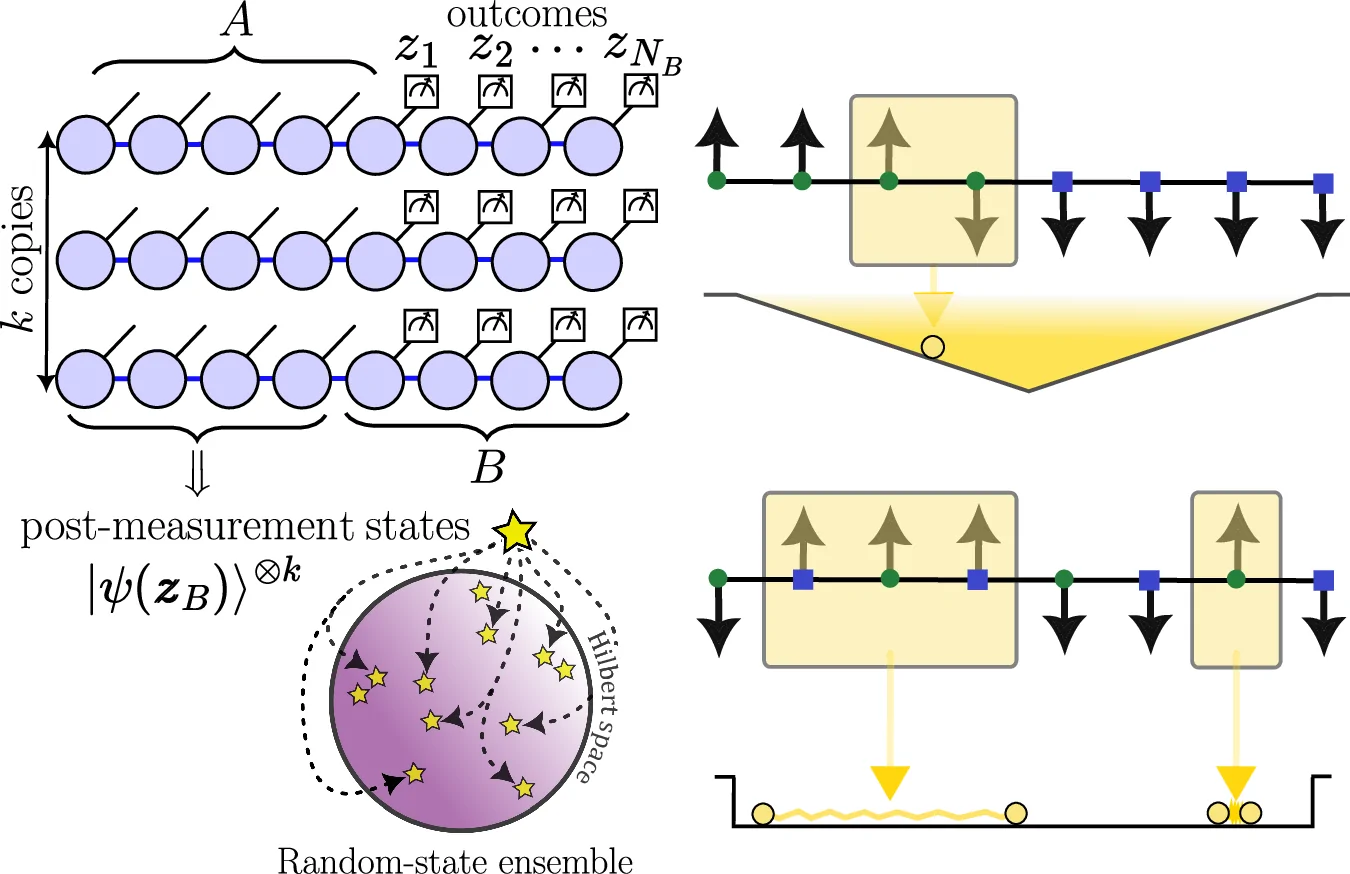
Sketch of the work. (Left) We consider a protocol where a random Matrix Product State (MPS) is prepared and subsequently subjected to projective measurements on the computational basis over a region *B*. The post-measurement states in the complementary region *A* are denoted $\left|\psi ({{\boldsymbol{z}}}_{B})\right\rangle$, where ***z***_*B*_ is the outcome string. We investigate the randomness of these states––specifically, how uniformly they sample the Hilbert space. (Upper right) The quantifier of randomness, the frame potential, is mapped into the evaluation of the partition function of an effective spin chain, whose degrees of freedom are permutations. The study of this model reveals an underlying structure: in the scaling limit, domain walls emerge as quasiparticles confined by an effective potential. (Lower right) We also consider a different geometry, in which MPSs are generated by a glued shallow circuit and sites *A* and *B* are alternated. In this setting, confinement also emerges in the form of a couple of domain walls bounded by a confining interaction.

The condition that the projected ensemble forms a *k*-design ${{\mathcal{F}}}_{{\mathcal{P}}[\left|\psi \right\rangle ]}^{(k)}={{\mathcal{F}}}_{{\rm{Haar}}}^{(k)}$ carries deep implications linking thermalization and eigenstate typicality across statistical mechanics, condensed-matter, and high-energy physics, revealing quantum complexity’s foundational role^40–44^. Characterizing the projected ensemble from a globally pure state $\left|\psi \right\rangle$ prepared with *limited resources* is challenging. Tensor-network states, as bounded-entanglement, tractable models, provide an ideal testbed. Here we study the projected ensemble for typical random one-dimensional tensor networks (MPS), offering a realistic, experimentally feasible route to random many-body states on a subsystem (see Fig. 1 for a sketch of the work).

### Random MPS and Glued circuits

Matrix product states (MPS) are one-dimensional tensor networks with bounded entanglement, defined by tensors ${M}_{\alpha \beta }^{(i)}({z}_{i})$ (with *α*, *β* ∈ {1,…,*χ*}, *z*_*i*_ ∈ {0, …, *d*−1})^12,13^. The amplitudes *ψ*(***z***) = 〈*z*_1_,…,*z*_*N*_∣*ψ*〉 follow from contracting these tensors. In diagrams, nodes denote tensors, lines their contractions, and open legs the physical indices^12^. For example, a generic MPS isScheme 1where black lines are physical qudits and blue lines are the bond indices of dimension *χ*.

We consider two protocols to generate projected ensembles from random MPS: both prepare the MPS via a random circuit and then measure a subset of qudits to define an ensemble on the remainder. As we shall show in the following, the two architectures lead to projected ensembles with *distinct emergent structures and randomness properties*. In particular, analyzing both protocols through the lens of the frame potential reveals two qualitatively different *confinement mechanisms* governing the buildup of randomness in the projected states. As a result, the deviation from the Haar distribution exhibits fundamentally different scaling behaviors with respect to the bond dimension *χ*.

*I. Staircase circuit*. The first protocol is based on the standard sequential construction of (random) matrix product states through *staircase circuits*^42,45–50^. Here, random gates ${U}^{(i)} \sim {\mathcal{U}}(d\chi )$ act sequentially on qudits initialized in $\left|0\right\rangle$, building the MPS. Measuring the last *N*_*B*_ qudits (including the final bond leg) projects onto an ensemble on *A*:Scheme 2

All qudits in *A* and *B* have dimension *d*, except the final site in *B* of dimension *χ*.

*II. Glued shallow circuits*. The second protocol uses a two-layer circuit: first, gates ${U}^{(i)} \sim {\mathcal{U}}(d{\chi }^{2})$ entangle each physical qudit with two auxiliaries; second, gates ${U}^{(i)} \sim {\mathcal{U}}({\chi }^{2})$ glue adjacent auxiliaries^51–57^. Measuring all auxiliaries in the computational basis defines the ensemble on the physical sites, which can be visually represented as:Scheme 3

This architecture results in a matrix product state with an efficient and parallelizable preparation scheme. The projected ensemble is defined on the *N*_*A*_ physical qudits of dimension *d*_*A*_ = *d*. In the second line of Scheme 3, we see that two adjacent auxiliary qudits can be combined into *N*_*B*_ = *N*_*A*_ + 1 *d*_*B*_-dimensional local Hilbert spaces undergoing projective measurements, with *d*_*B*_ = *χ*^2^.

Variants of this architecture have enabled rigorous results on the preparation of approximate quantum state designs with low-depth random circuits^58–61^. This work therefore provides a quantitative prediction for the measurement-induced randomness arising from their projected ensembles, clearly demonstrating the advantage of the glued circuit architecture for generating approximate quantum state designs.

### Mapping to a statistical model of permutations

In this section, we map the computation of the frame potential onto the partition function of an effective one-dimensional statistical model, whose degrees of freedom are permutations. The main steps are: (i) introducing a generalized frame potential amenable to the replica trick; (ii) linearizing the problem via Weingarten calculus on the replicated Hilbert space; (iii) identifying transfer matrices for sites in regions *A* and *B*; and (iv) reducing the partition function to a chain of *m*! × *m*! matrices, where *m* = 2(*n* + *k*) is the number of replicas.

We aim to compute the frame potential for the projected ensemble generated by the RMPS measurement protocols I and II. For *k* > 1, the negative exponents in Eq. (2) hinder analytic progress. To address this, we introduce the *generalized frame potential* defined as

3 $${{\mathcal{F}}}_{{\mathcal{P}}[\left|\psi \right\rangle ]}^{(k,n)}\equiv \mathop{\sum }\limits_{{{\boldsymbol{z}}}_{B},{{{\bf{z}}}^{{\boldsymbol{{\prime} }}}}_{B}}p{({{\boldsymbol{z}}}_{B})}^{n}p{({{{\boldsymbol{z}}}^{{\prime} }}_{{\rm{B}}})}^{n}| {\langle \widetilde{\psi }({{\boldsymbol{z}}}_{B})| \widetilde{\psi }({{{\boldsymbol{z}}}^{{\prime} }}_{{\rm{B}}})\rangle | }^{2k}.$$

The standard frame potential is recovered by setting *n* = 1 − *k*. To gain technical advantages and highlight general, state-independent features, we introduce an average over the global states $\left|\psi \right\rangle$ sampled over the RMPS ensemble, namely ${{\mathcal{F}}}^{(k,n)}\equiv {{\mathbb{E}}}_{\psi }\left[{{\mathcal{F}}}_{{\mathcal{P}}[\left|\psi \right\rangle ]}^{(k,n)}\right]$. The average ${{\mathbb{E}}}_{\psi }$ is equivalent to averaging over the random gates used in the circuits represented in Scheme 2, Scheme 3. With this convention, the standard (ensemble-averaged) frame potential reads ${{\mathcal{F}}}^{(k)}\equiv {{\mathcal{F}}}^{(k,1-k)}$. Using Haar invariance—specifically, the invariance under any single-qudit unitary *V* acting on *B*, which allows us to fix one measurement outcome—we can set ${\boldsymbol{z}}{\prime} \mapsto {{\bf{0}}}_{B}$ and ***z*** ≡ ***z***_*B*_, simplifying the generalized frame potential to ${{\mathcal{F}}}^{(k,n)}={D}_{B}{\sum }_{{\boldsymbol{z}}}{{\mathbb{E}}}_{\psi }\left[p{({\boldsymbol{z}})}^{n}\,p{({\bf{0}})}^{n}\,| \langle \widetilde{\psi }({\boldsymbol{z}})| \widetilde{\psi }({\bf{0}})\rangle {| }^{2k}\right]$. For general values of *n*, this expectation value over *ψ* remains problematic due to the non-linearity. To linearize the problem, we employ the *replica trick*, momentarily assuming that *n* is a positive integer and introducing *m* = 2(*n* + *k*) replicas of the system. This reformulation enables us to express the generalized frame potential as a linear functional on the replicated Hilbert space ${{\mathcal{H}}}^{\otimes m}$. We can rewrite the products of traces as a single trace by introducing suitable permutation operators *R*_*σ*_, where *σ* ∈ *S*_*m*_, which permute replicas as ${R}_{\sigma }| {x}_{1},{x}_{2},\ldots ,{x}_{m}\left.=\right|{x}_{{\sigma }^{-1}(1)},\ldots ,{x}_{{\sigma }^{-1}(m)}\rangle$. For example, for *m* = 2, we have ${\rm{tr}}(A){\rm{tr}}(B)={\rm{tr}}\left[(A\otimes B){R}_{()}\right]$ and ${\rm{tr}}(AB)={\rm{tr}}\left[(A\otimes B){R}_{(12)}\right]$, where *R*_()_ is the identity permutation and *R*_(12)_ is the swap operator, following standard cycle notation for permutations. In our case, defining ${\Lambda }^{(m)}={\sum }_{{\boldsymbol{z}}}{({I}_{A}\otimes \left|{\bf{0}}\right\rangle \left\langle {\boldsymbol{z}}\right|\otimes {I}_{A}\otimes \left|{\boldsymbol{z}}\right\rangle \left\langle {\bf{0}}\right|)}^{\otimes k}\otimes {({I}_{A}\otimes \left|{\boldsymbol{z}}\right\rangle \left\langle {\boldsymbol{z}}\right|\otimes {I}_{A}\otimes \left|{\bf{0}}\right\rangle \left\langle {\bf{0}}\right|)}^{\otimes n}$, Eq. (3) becomes

4 $$\begin{array}{l}{{\mathcal{F}}}^{(k,n)}={D}_{B}{{\mathbb{E}}}_{\psi }{\rm{tr}}\left[{\Lambda }^{(m)}\left|\psi \right\rangle {\left\langle \psi \right|}^{\otimes m}{R}_{{\sigma }_{A}}\right],\end{array}$$

where the permutation *σ*_*A*_ = (1, *k* + 1)(2, *k* + 2)⋯(*k*, 2*k*) captures the contraction pattern of the 2*k* replicas in $| \langle \widetilde{\psi }({\boldsymbol{z}})| \widetilde{\psi }(0)\rangle {| }^{2k}$, and is graphically represented byScheme 4

Due to the linearity of Eq. (4), the average over the RMPS ensemble can be brought inside the trace, reducing the problem to evaluating ${{\mathbb{E}}}_{\psi }\left[\left|\psi \right\rangle {\left\langle \psi \right|}^{\otimes m}\right]$, now a replica tensor network of bond dimension *χ*^2*m*^. This average over circuit realizations can be computed efficiently using the Weingarten calculus^28,62^, which expresses the Haar average of *m* copies of a unitary gate as a sum over permutation operators in *S*_*m*_. We work in the vectorized representation, where an operator *O* is mapped to $| O\rangle \rangle$, the conjugation *U*^†^*OU* becomes $({U}^{* }\otimes U)\,| O\rangle \rangle$, and the Hilbert-Schmidt inner product is expressed as ${\rm{tr}}({O}^{\dagger }V)=\langle \langle O| V\rangle \rangle$. When needed, we write $\,|V\rangle {\rangle }_{q}$ to emphasize the Hilbert space dimension. In this notation, the Haar average of *m* copies of a unitary gate reads

5 $${{\mathbb{E}}}_{U \sim {\mathcal{U}}(q)}\left[{({U}^{* }\otimes U)}^{\otimes m}\right]=\mathop{\sum }\limits_{\pi ,\sigma \in {S}_{m}}{{\rm{Wg}}}_{\pi ,\sigma }(q)\,|\sigma\rangle\rangle\langle \,\langle \pi|,$$

where we use the shorthand $|\sigma\rangle\rangle \equiv|{R}_{\sigma }\rangle\rangle$ to denote the vectorized representation of the permutation operator *R*_*σ*_. In the relevant case where $U \sim {\mathcal{U}}(d\chi )$ acts on a product Hilbert space, the permutation operators decompose into product form ${|\sigma\rangle\rangle }_{d\chi }={|\sigma\rangle\rangle }_{d}\otimes {|\sigma\rangle\rangle }_{\chi }$. Importantly, the permutation representations are not orthonormal: their overlaps define the Gram matrix 〈〈*σ*∣*π*〉〉_*q*_ = : *G*_*σ*,*π*_(*q*) = *q*^*m*−d(*σ*, *π*)^ where ${\mathtt{d}}(\sigma ,\sigma {\prime} )$ is the transposition distance, i.e., the minimum number of elementary transpositions required to transform *σ* into $\sigma {\prime}$.

To proceed, we exploit the structure of the RMPS state. Recall that ${I}_{A}={I}^{\otimes {N}_{A}}$ and ${R}_{{\sigma }_{A}}={r}_{{\sigma }_{A}}^{\otimes N}$, where the permutations *I* and ${r}_{{\sigma }_{A}}$ act on the replicas of a single qudit. Moreover, the measurement projector can be written as a product over qudit projectors $\left|{\boldsymbol{z}}\right\rangle \left\langle {\boldsymbol{z}}\right|{=\bigotimes }_{j=1}^{{N}_{B}}\left|{z}_{j}\right\rangle \left\langle {z}_{j}\right|$, where *z*_*j*_ ∈ {0,…,*d*_*B*_ − 1}. This tensor-product structure enables the identification of two key transfer matrices, each arising from the average over the local MPS tensor and acting on the replicas of a single physical site (see Eq. (4); a detailed derivation is provided in the Methods, Sec. Computations of the Transfer Matrices). For sites *i* ∈ *A*, the identity in *Λ*^(*m*)^ acts trivially; hence the nontrivial structure arises from the contraction with the permutation operator ${r}_{{\sigma }_{A}}$. This contraction can be represented graphically by focusing on a single MPS tensor from Scheme 1, replicating it and its conjugate *m* times, and inserting ${r}_{{\sigma }_{A}}$ in between, yielding:Scheme 5

Here, darker shapes represent complex conjugate tensors. Then, using the Weingarten formula, we can perform directly the average and obtain

6 $${\widetilde{{\mathcal{T}}}}_{A}=\mathop{\sum }\limits_{\pi ,\sigma }{|\pi\rangle\rangle }_{\chi }{{\rm{Wg}}}_{\pi ,\sigma }({d}_{A}\chi ){\upsilon }_{\sigma }^{(j)}({d}_{A}){\langle\langle \sigma|}_{\chi},$$

where ${\upsilon }_{\sigma }^{(j)}({d}_{A})={G}_{\sigma ,{\sigma }_{A}}({d}_{A})$ for *j* ∈ *A* and *σ*_*A*_ ∈ *S*_*m*_ is the permutation in Scheme 4. For sites *i* ∈ *B*, instead, the permutation ${r}_{{\sigma }_{A}}$ swaps $\left|{z}_{i}\right\rangle$ with $\left|{0}_{i}\right\rangle$. This implies that in region *B* the MPS tensors (replicated *m* times) are contracted with the operator $d{\sum }_{{\boldsymbol{z}}}{(\left|{\boldsymbol{z}}\right\rangle \left\langle {\boldsymbol{z}}\right|\otimes \left|{\bf{0}}\right\rangle \left\langle {\bf{0}}\right|)}^{\otimes (n+k)}$. Graphically, we therefore obtain:Scheme 6

For the Weingarten formula, one needs to evaluate ${\upsilon }_{\sigma }^{(j)}({d}_{B})={d}_{B}{\sum }_{{z}_{j}}{\rm{tr}}[{(|{z}_{j}\rangle \langle {z}_{j}|\otimes |0\rangle \langle 0|)}^{\otimes (m/2)}{R}_{\sigma }]$. For *z*_*j*_ = 0, all permutations contribute the same value *d*_*B*_. In contrast, when *z*_*j*_ ≠ 0, any permutation that contracts a $\left|{z}_{j}\right\rangle$ with a $\left|0\right\rangle$ results in zero contribution to the trace. This mechanism favors the subset ${S}_{m}^{{\rm{fact}}}:={S}_{m/2}\times {S}_{m/2}$, consisting of permutations that independently permute the ${\left|{z}_{j}\right\rangle }^{\otimes m/2}$ and ${\left|0\right\rangle }^{\otimes m/2}$ replicas among themselves without mixing. To capture this structure, we introduce the indicator function 1_*F*_(*σ*), which is 1 if $\sigma \in {S}_{m}^{{\rm{fact}}}$ and 0 otherwise. This yields ${\upsilon }_{\sigma }^{(i)}(q)={q}^{2}{1}_{F}(\sigma )+q(1-{1}_{F}(\sigma ))$, and the resulting transfer matrix

7 $$\begin{array}{l}{\widetilde{{\mathcal{T}}}}_{B}=\mathop{\sum }\limits_{\pi ,\sigma }{|\pi\rangle\rangle }_{\chi }{{\rm{Wg}}}_{\pi ,\sigma }({d}_{B}\chi ){\upsilon }_{\sigma }^{(j)}({d}_{B}){\langle\langle \sigma|}_{\chi }.\end{array}$$

Finally, the boundary conditions of the original matrix product state translate into left $\langle\langle {\mathcal{L}}|$ and right $|{\mathcal{R}}\rangle\rangle$ boundary vectors in the replica tensor network. For example, in protocol I, the boundary state $\left\langle 0\right|$ becomes $\langle \langle {\mathcal{L}}|={\langle\langle (|0\rangle \langle 0|)|}^{\otimes m}$. Putting all elements together, the generalized frame potential ${{\mathcal{F}}}^{(k,n)}$ reduces to

8 $${{\mathcal{F}}}^{(k,n)}=\langle \langle {\mathcal{L}}| {\widetilde{{\mathcal{T}}}}_{1}{\widetilde{{\mathcal{T}}}}_{2}\ldots {\widetilde{{\mathcal{T}}}}_{N-1}| {\mathcal{R}}\rangle \rangle ,$$

where ${\widetilde{{\mathcal{T}}}}_{j}$ is either ${\widetilde{{\mathcal{T}}}}_{A}$ or ${\widetilde{{\mathcal{T}}}}_{B}$ depending on the location *j*.

The final step of the mapping involves contracting both the boundary and bulk indices of the transfer matrices ${\widetilde{{\mathcal{T}}}}_{j}$, which have bond dimension *χ*^*m*^. This reduces the problem to a computation in a vector space of dimension *m*!, spanned by the orthonormal basis $\{| \left.\pi \right),\pi \in {S}_{m}\}$, independent of *χ*. To proceed, we first observe that contractions between consecutive transfer matrices ${\widetilde{{\mathcal{T}}}}_{j}$ introduce additional factors from the Gram matrix *G*_*π*,*σ*_(*χ*). To incorporate these contributions, we define new transfer matrices ${[{{\mathcal{T}}}_{i}]}_{\sigma ,\sigma {\prime} }={T}_{\sigma ,\sigma {\prime} }{\upsilon }_{\sigma }^{(i)}(d)$ of size *m*! × *m*!, where ${T}_{\sigma ,\sigma {\prime} }={{\rm{Wg}}}_{\sigma ,\sigma {\prime} }({d}_{j}\chi ){G}_{\sigma ,\sigma {\prime} }(\chi )$, with *d*_*j*={*A*, *B*}_ the local Hilbert space dimension. Under this convention, the left boundary vector in the reduced *m*!-dimensional space is given by $({v}_{L}| \pi )\equiv \langle \langle {\mathcal{L}}| \pi \rangle \rangle$, while the right boundary vector is $(\pi | {v}_{R})\equiv {\sum }_{\sigma }{{\rm{Wg}}}_{\sigma ,\pi }\langle \langle \sigma | {\mathcal{R}}\rangle \rangle$. Collecting all ingredients, we arrive at a statistical mechanical formulation of ${{\mathcal{F}}}^{(k,n)}$ as a partition function over *m*!-dimensional degrees of freedom

9 $$\begin{array}{l}(k,n)\,=\,({v}_{L}| {{\mathcal{T}}}_{1}{{\mathcal{T}}}_{2}\ldots {{\mathcal{T}}}_{N-1}| {v}_{R})=\\ \,\,\,\,\,\,\,=\,\mathop{\sum }\limits_{{\sigma }_{1},\ldots ,{\sigma }_{N}}{v}_{L}({\sigma }_{1})\left(\mathop{\prod }\limits_{a=1}^{N-1}{T}_{{\sigma }_{a},{\sigma }_{a+1}}{\upsilon }_{{\sigma }_{a}}^{(a)}\right){v}_{R}({\sigma }_{N}).\end{array}$$

In essence, the computation of the frame potential reduces to evaluating the partition function of an effective one-dimensional Ising-like model, where the degrees of freedom are permutations in *S*_*m*_.

So far, our analysis remains general and applies to a broad class of ensembles. The specific choice of ensemble is encoded in the form of the Weingarten symbol. For Haar-random unitaries, Wg = *G*^−1^ corresponds to the (pseudo)inverse of the Gram matrix. In contrast, for a Gaussian ensemble with variance *ς*, the Weingarten symbol takes the diagonal form Wg_*π*,*σ*_ = *ς*^2*m*^*δ*_*π*,*σ*_ reflecting Wick’s theorem ^63–66^; see Methods for details. With these definitions, setup I corresponds to the choice *A* = {1,…,*N*_*A*_}, while setup II uses the alternating pattern *A* = {1, 3,…,*N* = 2*N*_*A*_ − 1} (with minor differences in the dimension parameter *d*_*B*_, see Methods for details). Evaluating Eq. (9) analytically for arbitrary *k* and *n* remains challenging in general, yet it is necessary in order to perform the replica limit *n* → 1 − *k*. However, the structure of the statistical model is transparent: the matrix ${T}_{\sigma ,\sigma {\prime} }(\chi ,d)$ plays the role of a two-site interaction, while ${\upsilon }_{\sigma }^{(i)}$ acts as an on-site potential, whose form depends on whether the site *i* belongs to the region *A* or *B*.

#### Low-temperature expansion

In the partition function Eq. (9), the bond dimension *χ* plays the role of a sort of ‘inverse temperature’ from the statistical mechanics perspective, as it controls the strength of the effective ferromagnetic interactions, suppressing domain-wall excitations as *χ* increases. Our main strategy for quantifying randomness generation at arbitrary replica number *m* = 2(*n* + *k*)—enabling analytic continuation to the replica limit *n* → 1 − *k*—is to perform a low-temperature (large *χ*) expansion of Eq. (9) induced by Haar-random gates

10 $${{\mathcal{F}}}^{(n,k)}=\mathop{\sum }\limits_{\alpha \ge 0}{{\mathcal{F}}}^{(n,k,\alpha )},$$

where each term scales as ${{\mathcal{F}}}^{(n,k,\alpha )}=O({\chi }^{-\alpha })$. To carry out this expansion, we express the interaction matrix *T* in powers of 1/*χ*. Using the adjacency matrices between permutations, defined as ${{\mathsf{A}}}_{\sigma \pi }^{(\alpha )}={\delta }_{{\mathtt{d}}(\sigma ,\pi ),\alpha }$, we have

11 $${G}_{\sigma \pi }(\chi )={\chi }^{m}\left({\delta }_{\sigma \pi }+\mathop{\sum }\limits_{\alpha \ge 1}{\chi }^{-\alpha }{{\mathsf{A}}}_{\sigma \pi }^{(\alpha )}\right),$$

12 $${{\rm{Wg}}}_{\sigma \pi }(d\chi )={(d\chi )}^{-m}{\left({\delta }_{\sigma \pi }+\mathop{\sum }\limits_{\alpha \ge 1}{(d\chi )}^{-\alpha }{{\mathsf{A}}}_{\sigma \pi }^{(\alpha )}\right)}^{-1}$$

13 $${T}_{\sigma ,\pi }(\chi ,d)={d}^{-m}({\delta }_{\sigma ,\pi }+\mathop{\sum }\limits_{\alpha \ge 1}{{\mathsf{T}}}_{\sigma ,\pi }^{(\alpha )}\,{\chi }^{-\alpha }).$$

As we will see, while the leading-order contribution is universal, the subleading corrections—and hence the emergent physics—depend sensitively on the geometry, and in particular on the choice of setup. We note that, unlike in scaling limits where *N*/*χ* is held fixed (as in Refs. ^50,66,67^), the corrections ${{\mathsf{T}}}^{(\beta \, > \,1)}$ remain relevant in the present setting, because the domain walls are confined and can overlap on the same bond with *O*(1) probability. The leading contribution *α* = 0 corresponds to the limit ${G}_{\sigma ,\sigma {\prime} }(\chi )\mathop{=}\limits^{\chi \to \infty }{\chi }^{m}{\delta }_{\sigma ,\sigma {\prime} }$ and similarly ${\rm{Wg}}(d\chi )\simeq {(d\chi )}^{-m}{\delta }_{\sigma ,\sigma {\prime} }$ is fixed by the pseudo inverse condition on *G*(*dχ*). It follows that ${T}_{\sigma ,\sigma {\prime} }(\chi ,d)\mathop{=}\limits^{\chi \to \infty }{d}^{-m}{\delta }_{\sigma ,\sigma {\prime} }$, implying a strong ferromagnetic coupling that propagates a fixed permutation through the chain, modulated only by the on-site potentials ${\upsilon }_{\sigma }^{(i)}$. Moreover, measurements on *O*(*χ*)-dimensional qudits constrain the dominant contributions to permutations $\pi \in {S}_{m}^{{\rm{fact}}}$. For example, in setup I, the right boundary becomes $(\sigma | {v}_{R})={\sum }_{\pi }{{\rm{Wg}}}_{\sigma ,\pi }(d\chi ){\upsilon }_{\pi }^{({N}_{B})}(d\chi )\simeq {(d\chi )}^{2-m}{1}_{F}(\sigma )$, with a similar behavior in setup II. Collecting these remarks, for *χ* → *∞*, we have

14 $${{\mathcal{F}}}^{(k,n,0)}\mathop{=}\limits^{\chi \gg 1}\frac{{d}^{-m{N}_{A}}}{{(\chi {d}^{{N}_{B}})}^{(m-2)}}\mathop{\sum }\limits_{\sigma \in {S}_{m}^{{\rm{fact}}}}{[{\upsilon }_{\sigma }^{(A)}]}^{{N}_{A}}.$$

At large *N*_*A*_, the dominant contributions arise from permutations that minimize the distance to *σ*_*A*_. For any *n*, there are exactly *k*! such permutations, of the form *σ* = *τ*_1_*τ*_2_ ⋯ *τ*_*k*_*σ*_*A*_, with {*τ*_*i*_} a set of *k* commuting transpositions, thus satisfying d(*σ*, *σ*_*A*_) = *k*^38,39^. Taking the replica limit *n* → 1 − *k* (so *m* → 2), Eq. (14) simplifies to the Haar frame potential for subsystem *A*: ${{\mathcal{F}}}^{(k,1-k,0)}={{\mathcal{F}}}_{{\rm{Haar}}}^{(k)}=k!{D}_{A}^{-k}$. Thus, in the *χ* → *∞* limit, the projected ensemble generates maximally random states in subsystem *A*. The factor *k*! reflects the ground-state degeneracy associated with the *k*! permutations minimizing the distance to *σ*_*A*_ (see Methods for further discussion). At finite *χ*, however, the off-diagonal terms in *T*(*χ*, *d*) allow domain walls—interfaces between different permutations—to emerge. The effect of these excitations will be explored in the next two sections.

### Confinement mechanisms

We now turn to the mechanism of confinement underlying randomness generation in the two setups. We begin with a detailed analysis of the staircase circuit (setup I), followed by the glued circuit geometry (setup II). Additional technical details, including intermediate steps for the simplified Gaussian ensemble, are provided in the Methods.

#### Confinement for RMPS obtained through staircase circuits

Recall that setup I is characterized by the partition function ${{\mathcal{F}}}^{(k,n)}=({v}_{L}| {{\mathcal{T}}}_{A}^{{N}_{A}}{{\mathcal{T}}}_{A}^{{N}_{B}}| {v}_{R})$, with boundary (*v*_*L*_∣*σ*) = 1 for any *π* and $(\sigma | {v}_{R})={\sum }_{\pi }{{\rm{Wg}}}_{\sigma ,\pi }{\upsilon }_{\pi }^{({N}_{B})}(d\chi )$. Thus, corrections in 1/*χ*—arising from domain wall formation—are controlled by the expansion of *T* in Eq. (13). The first term of this expansion (*α* = 1), reads ${{\mathsf{T}}}^{(1)}=\frac{d-1}{d}{{\mathsf{A}}}^{(1)}$ and encodes nearest-neighbor permutation hoppings. The first-order correction corresponds to *α* = 1 in Eq. (10), and arises by replacing a single *T*(*χ*, *d*) in the product of Eq. (9) with the adjacency matrix ${{\mathsf{T}}}^{(1)}$, while keeping all other factors proportional to *δ*-functions. As a result, the matrix element ${{\mathsf{T}}}_{\sigma {\prime} ,\sigma }^{(1)}$ on a certain bond *j* is associated with a domain wall, so that all on-site potentials to the right are evaluated on *σ*, while those to the left in $\sigma {\prime}$. This fact, together with the previously discussed restriction $\sigma \in {S}_{m}^{{\rm{fact}}}$ imposed by the rightmost spin in *B*, leads to

15 $${{\mathcal{F}}}^{(n,k,1)}={d}^{-mN}{d}^{2}{\chi }^{2-m}\mathop{\sum }\limits_{{{{\sigma}^{\prime}},\sigma}\atop{\sigma \in {S}_{m}}}^{{\rm{fact}}}\frac{1}{\chi }{{\mathsf{T}}}_{\sigma {\prime} ,\sigma }^{(1)}\mathop{\sum }\limits_{j=1}^{N-1}\mathop{\prod }\limits_{i=1}^{j}{\upsilon }_{\sigma {\prime} }^{(i)}\mathop{\prod }\limits_{r=j+1}^{N-1}{\upsilon }_{\sigma }^{(r)}.$$

We analyze Eq. (15) in the regime *N*_*B*_ ≫ *N*_*A*_ ≫ 1, interested in how the factor 1/*χ* can be offset by the possible choices of $\sigma {\prime}$. Since factorized permutations already maximize onsite contributions in *B*, to compensate for the 1/*χ* factor, the only possibility is for the permutation $\sigma {\prime}$ to be closer to *σ*_*A*_. Indeed, in this case, starting with *σ* = *τ*_1_…*τ*_*k*_*σ*_*A*_ as one of the *k*! ground states at distance *k* from *σ*_*A*_, there are precisely *k* choices $\tau \in {\{{\tau }_{i}\}}_{i}$ that make ${\mathtt{d}}(\sigma {\prime} =\tau \sigma ,{\sigma }_{A})=k-1$. Comparing Eq. (15) with (14), this results in a factor ${\upsilon }_{\sigma {\prime} }^{(A)}/{\upsilon }_{\sigma }^{(A)}=d$ for each site in *A* where such a $\sigma {\prime}$ replaces *σ*. Finally, the sum over *j*, i.e., the location of the domain wall, must be addressed. It is convenient to consider the possible positions of the domain wall relative to the interface between *A* and *B*, i.e., *ℓ* = *j* − *N*_*A*_. First, if *ℓ* < 0, the domain wall is inside region *A* and the gain due to $\sigma {\prime}$ replacing *σ* equals ${d}^{{N}_{A}-| \ell | }$. Conversely, if *ℓ* > 0, the domain wall is inside region *B* and the entire region *A* is covered by $\sigma {\prime}$ resulting in a gain ${d}^{{N}_{A}}$. However, since $\sigma {\prime}$ cannot be factorized, there is a suppression factor ${\upsilon }_{\sigma {\prime} }^{(B)}/{\upsilon }_{\sigma }^{(B)}={d}^{-1}$ for each site in *B* where $\sigma {\prime}$ replaces *σ*. From this argument, we see that the natural scaling variable in the limit *N*_*B*_ ≫ *N*_*A*_ ≫ 1 and *χ* → *∞* is

16 $$x=\frac{{D}_{A}}{\chi }\frac{d-1}{d}=\frac{{d}^{{N}_{A}}}{\chi }\frac{d-1}{d},$$

where we inserted for convenience the factor (*d* − 1)/*d* arising from the hopping matrix ${{\mathsf{T}}}^{(1)}$. We will denote as “$\mathop{=}\limits^{{\rm{s.l.}}}$” equalities in the scaling limit at fixed *x*. Taking into account the two signs of *ℓ*, one has

17 $${{\mathcal{F}}}^{(n,k,1)}\mathop{=}\limits^{{\rm{s.l.}}}{{\mathcal{F}}}^{(n,k,0)}\,xk\mathop{\sum }\limits_{\ell =-\infty }^{\infty }{d}^{-| \ell | }={{\mathcal{F}}}^{(n,k,0)}\,xk\frac{d+1}{d-1}.$$

Note that in the scaling limit considered, both *N*_*A*_, *N*_*B*_ → *∞*, which allowed us to extend the geometric sum over *ℓ* to infinity. In practice, Eq. (17) can be interpreted as the partition function of a domain wall confined by a potential of strength ∝ ∣*ℓ*∣, where *ℓ* denotes the position. This result admits a natural interpretation in the language of quantum circuits with depth growing in time *t*. In such circuits, the bond dimension scales exponentially with time as $\chi ={e}^{{v}_{E}t}$, where *v*_*E*_ is the entanglement velocity^63^. Taking the replica limit *n* → 1 − *k*, the first-order expansion of the frame potential becomes

18 $${{\mathcal{F}}}^{(k)} \sim k!{d}^{-{N}_{A}}\left(1+xk\frac{d+1}{d-1}\right)=k!\,\left({d}^{-{N}_{A}}+\frac{k!(d+1)}{d}{e}^{-{v}_{E}t}\right),$$

where in the second expression, we used the explicit form of *x* from Eq. (16). This form mirrors the minimal membrane picture that emerges in random circuits^63^: the two terms correspond to configurations in which the membrane exits either through the left boundary of the system (with cost *O*(*N*_*A*_)) or through the bottom boundary (with cost *O*(*t*))^39,68^. Heuristically, these two kinds of membrane trajectories can be represented as followsScheme 7

Thus, the statistical mechanics of the random tensor network faithfully reproduces the competing pathways of membrane dynamics in circuit-based models. As we will see in the next section, however, such a membrane picture cannot be effectively applied to the second architecture we consider (Setup II).

**Fig. 2:**
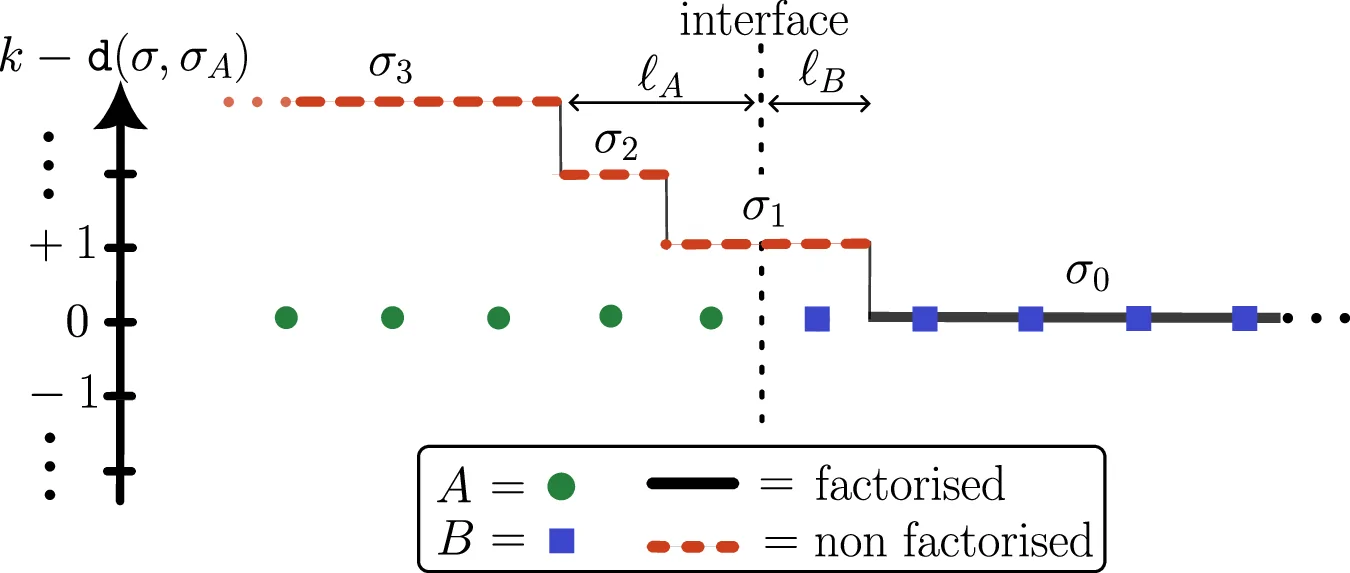
Graphical representation of domain wall excitations and confinement in staircase RMPS (setup I), with the y-axis indicating the difference *k* − d(*σ*, *σ*_*A*_). We depict dominant contributions in the scaling limit at order *α* in the 1/*χ* expansion of the frame potential, i.e., ${{\mathcal{F}}}^{(n,k,\alpha )}$ (here with *α* = 3).

We are now in a position to analyze higher-order corrections in the 1/*χ* expansion. These are governed by multiple insertions of the matrices ${{\mathsf{T}}}^{(\beta )}$ for *β*≥1, but for clarity, we continue focusing first on *β* = 1. At order *α*, the contribution ${{\mathcal{F}}}^{(n,k,\alpha )}$ involves *α* insertions of the matrix ${{\mathsf{T}}}^{(1)}$, corresponding to *α* elementary domain walls (i.e., transpositions). This leads to a configuration composed of *α* + 1 domains, labeled *σ*_*α*_, *σ*_*α*−1_, …, *σ*_1_, *σ*_0_ = *σ*, with $\sigma \in {S}_{m}^{{\rm{fact}}}$. From the scaling behavior in Eq. (16), it follows that to offset the *χ*^*α*^ suppression, the leftmost domain must be within distance d(*σ*_*α*_, *σ*_*A*_) = *k* − *α* and must cover nearly the entire region *A* (i.e., *N*_*A*_ − *O*(1) sites). This enforces a rigid domain structure: proceeding from right to left, each domain wall reduces the permutation’s distance to *σ*_*A*_ by exactly one: d(*σ*_*a*_, *σ*_*A*_) = *k* − *a*, as illustrated in Fig. 2.

Given a ground state of the form *σ*_0_ = *τ*_1_…*τ*_*k*_*σ*_*A*_, the *α* domain walls correspond to selecting *α* transpositions from the set *τ*_*i*_, in a specific order. This leads to a combinatorial factor of *k*!/(*k* − *α*)! possible ordered sequences. We also briefly address the positioning of the domain walls, denoted by *ℓ*_*α*_ < *ℓ*_*α*−1_ < … < *ℓ*_1_. As clarified earlier, domain walls in *A* (*ℓ*_*i*_ < 0) produce progressive approaches to *σ*_*A*_. In contrast, in *B*, any *σ*_*a*_ ≠ *σ* will not be factorized and encounters the same 1/*d* suppression factor. This induces an asymmetric potential in the two regions

19 $$V({\ell }_{1},\ldots ,{\ell }_{\alpha })={V}_{A}({\ell }_{1},\ldots ,{\ell }_{\alpha })+{V}_{B}({\ell }_{1},\ldots ,{\ell }_{\alpha }),$$

with the explicit forms (see Methods for the detailed derivation)

20 $${V}_{A}({\ell }_{1},\ldots ,{\ell }_{\alpha })=\mathop{\sum }\limits_{i=1}^{\alpha }\max (-{\ell }_{i},0)$$

21 $${V}_{B}({\ell }_{1},\ldots ,{\ell }_{\alpha })=\max ({\ell }_{1},\ldots ,{\ell }_{\alpha },0).$$

Finally, we turn to subleading corrections governed by ${{\mathsf{T}}}^{(\beta )}$ for *β* > 1. These correspond to configurations in which multiple domain walls occur at the same bond, i.e., *ℓ*_*i*_ = *ℓ*_*i*+1_ = … = *ℓ*_*i*+*β*−1_. The matrices ${{\mathsf{T}}}^{(\beta )}$ encode the interaction between overlapping domain walls. This interaction mechanism marks a key distinction from the scaling limits studied in Refs. ^50,66,67^, where the thermodynamic limit with *N*/*χ* = fixed leads to dilute domain walls. In that regime, all ${{\mathsf{T}}}^{(\beta \, > \,1)}$ became irrelevant, giving rise to universal behavior. By contrast, in our setting, these interaction terms remain relevant. Nevertheless, for Haar RMPS the problem remains tractable since domain walls are associated with transpositions that commute. Specifically, placing *p* commuting transpositions on the same bond yields a contribution ${{\mathsf{T}}}_{{\tau }_{1}\ldots {\tau }_{p},{\bf{1}}}^{(p)}={((d-1)/d)}^{p}$ which can be conveniently absorbed into the definition of the scaling variable *x* in Eq. (16) (see Methods). In the scaling limit Eq. (16), this leads to the compact expression

22 $${{\mathcal{F}}}^{(n,k,\alpha )}\mathop{=}\limits^{{\rm{s.l.}}}{{\mathcal{F}}}^{(n,k,0)}\left(\begin{array}{c}k\\ \alpha \end{array}\right){x}^{\alpha }\mathop{\sum }\limits_{{\ell }_{1},\ldots ,{\ell }_{\alpha }=-\infty }^{\infty }{d}^{-V({\ell }_{1},\ldots ,{\ell }_{\alpha })}$$

**Fig. 3:**
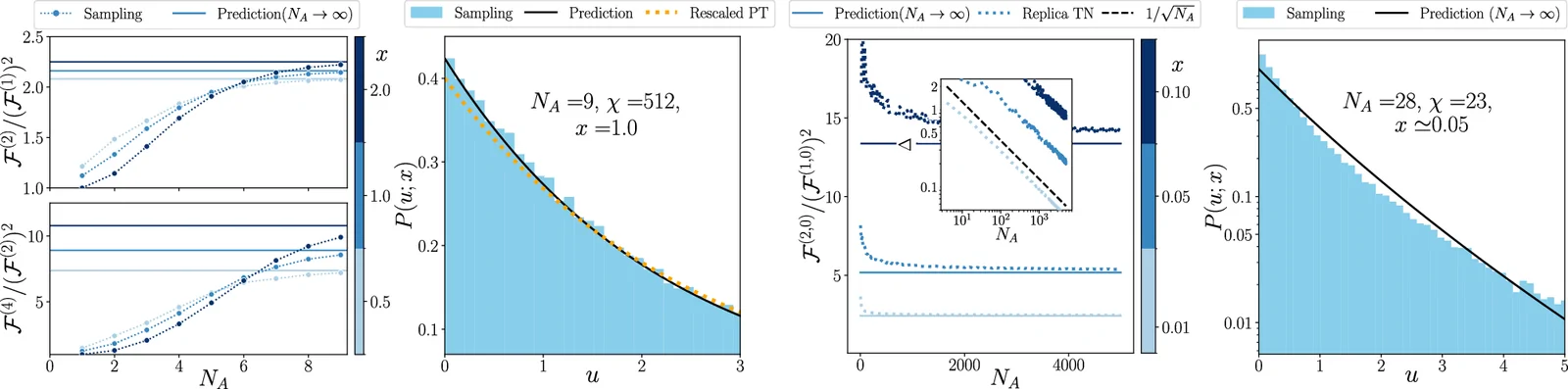
Staircase RMPS (Setup I). (Left). Ratios of the moments ${{\mathcal{F}}}^{(k)}$ for the projected ensemble. Numerical results obtained via Born rule sampling of random MPS (1500 measurements per state, averaged over 100 realizations) are compared to analytical predictions (horizontal lines), as a function of subsystem size *N*_*A*_. The MPS bond dimension scales as $\chi ={2}^{{N}_{A}}/x$, with ${N}_{B}=\lfloor {N}_{A}^{3/2}\rfloor$. (Mid-left). Distribution *P*(*u*; *x*) of the overlaps *u* for *N*_*A*_ = 9, *χ* = 512, corresponding to *x* = *D*_*A*_/*χ* = 1.0. The histogram from Born rule sampling of random MPS (same data as in (plot Left)) is compared with the analytical prediction for *P*(*u*; *x*) (black line) and a rescaled Porter-Thomas (PT) distribution (orange dotted line). The analytical result captures the *u* = 0 behavior, unlike the naive PT ansatz. **Glued shallow circuit RMPS (Setup II)**. (Mid-Right). Ratios of generalized frame potentials ${{\mathcal{F}}}^{(k,n)}$ for *n* = 0 (forced bitstring sampling). Here $\chi =\lfloor \sqrt{{N}_{A}/x}\rfloor$. Results from exact replica tensor network contractions (dotted lines) are compared to our analytical predictions (horizontal lines), valid in the *N*_*A*_ → *∞* limit. Convergence is assessed via the discrepancy $\Delta ={\left.{{\mathcal{F}}}^{(2,0)}/{({{\mathcal{F}}}^{(1,0)})}^{2}\right|}_{{\rm{TN}}}-{\left.{{\mathcal{F}}}^{(2,0)}/{({{\mathcal{F}}}^{(1,0)})}^{2}\right|}_{{\rm{Pred}}}$ (inset), which decays as $1/\sqrt{{N}_{A}}$ (black dotted line). (Right). Distribution *P*(*u*; *x*) for *N*_*A*_ = 28, *χ* = 23, corresponding to *x* ≃ 0.05 ≃ *N*_*A*_/*χ*^2^. The histogram from Born rule sampling is compared to the analytical prediction (black line). Due to small system sizes, finite-size effects limit quantitative agreement.

Summing over all orders *α*, the replica-limit frame potential becomes

23 $${{\mathcal{F}}}^{(k)}(x)={{\mathcal{F}}}_{{\rm{Haar}}}^{(k)}\frac{d-1}{d}\mathop{\sum }\limits_{j=0}^{+\infty }{d}^{-j}{\left(1+x\frac{d}{d-1}+xj\right)}^{k}\,,$$

where the sum over the discrete variable *j* is reminiscent of the fact that the confining length *ℓ*_*A*_, *ℓ*_*B*_ can only take integer values. The expression Eq. (23) recovers previous results^38,39^ in the limit of large qudit dimension *d* ≫ 1, where the sum reduces to ${{\mathcal{F}}}^{(k)}(x)\approx {{\mathcal{F}}}_{{\rm{Haar}}}^{(k)}{(1+x)}^{k}$. However, our formula holds for arbitrary qudit dimension *d* and explicitly reveals how finite *d* induces discrete fluctuations in the confining interface. In Fig. 3, we compare this analytical prediction for the frame potential at small *k* with numerical simulations from random MPS samples. The agreement is excellent—even for moderate values of *N*_*A*_. Moreover, the analysis remains robust as long as the bath size grows polynomially faster than the region *A*: that is, ${N}_{B} \sim {N}_{A}^{\nu }$ with exponent *ν* > 1. This shows that a relatively small bath suffices to induce near-Haar randomness in *A* up to a finite precision. Finally, note that in deriving Eq. (23), we took the scaling limit before the replica limit, whereas, formally, the correct order of limits should be the reverse. Nevertheless, numerical results strongly support the validity of our final expressions. Moreover, for generic finite replicas, the scaling limit we identify exists, with corrections that are subleading in *N*.

#### Confinement of glued shallow circuits RMPS

**Fig. 4:**
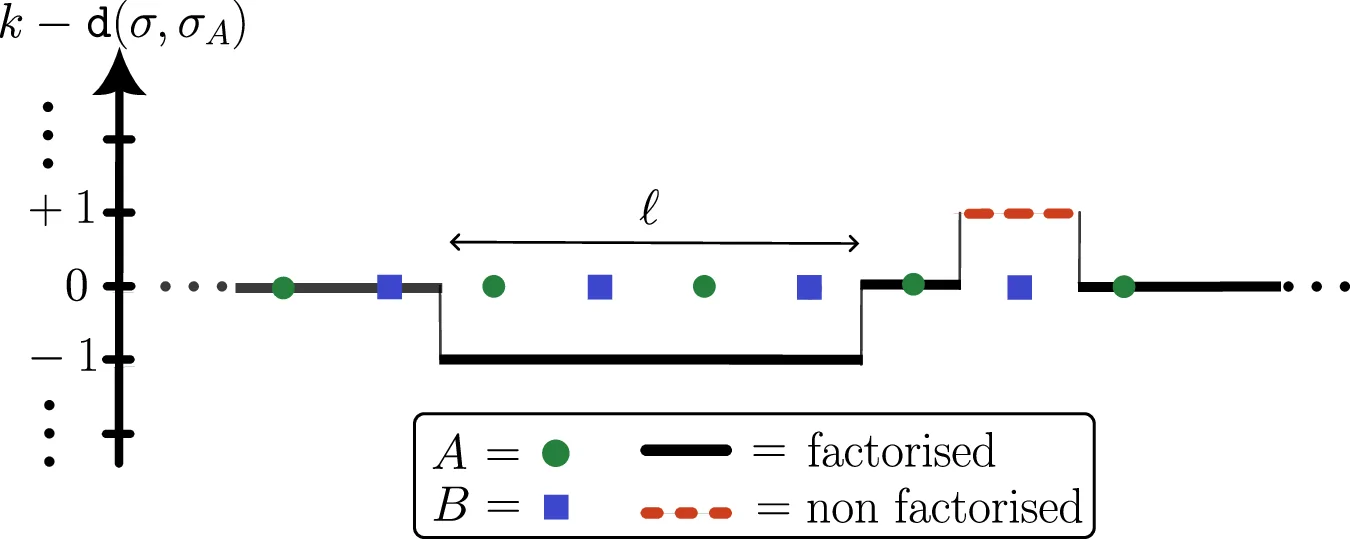
Graphical representation of domain wall excitations and confinement in the glued shallow circuit RMPS (setup II), with the y-axis indicating the difference *k* − d(*σ*, *σ*_*A*_). We depict dominant contributions in the scaling limit at order *α* = 4 in the 1/*χ* expansion of the frame potential.

As in the previous case, excitations can appear at cost 1/*χ* per domain wall. However, since sites *A* and *B* are now alternating and the local dimension on *B* sites is *χ*^2^, the nature of excitations changes significantly. As before, a domain wall may be created to bring the permutation closer to *σ*_*A*_ on a site *A*. However, this process necessarily creates a non-factorized permutation. Because such permutation incur an energy cost of 1/*χ*^2^ at every *B* site, the resulting excitations must be annihilated by a compensating domain wall that restores the original factorized permutation without including any site in *B* (see dashed red line in Fig. 4). Overall, these kink-antikink excitations therefore occur at a cost of 1/*χ*^2^, and since they can appear on all *A* sites, their natural scaling variable is

24 $$x=\frac{{N}_{A}}{{\chi }^{2}}.$$

Consequently, we will consider the scaling limit where both *N*_*A*_ and *χ* are large while *x* remains fixed. In this limit, additional excitations with the same scaling behavior emerge. For instance, at any point of the chain the current permutation can ‘jump’ to another factorized permutation at distance d(*σ*, *σ*_*A*_) = *k* + 1 via a domain wall, persist in this configuration over *ℓ* sites, and subsequently revert to the original state. Crucially, the domain wall pairs associated with these excitations experience a confining interaction potential, as their contribution is suppressed by a factor 1/*d* for each site *A* involved, a quantity scaling linearly with *ℓ* (see black thick line in Fig. 4). Thus, these excitations are meson-like with a *O*(1) extension and can be created on all sites, giving a scaling consistent with Eq. (24).

In Figure 4, *ℓ* is the confinement length and the amplitude associated to a finite *ℓ* scales as *d*^−*ℓ*^. Our analysis of setup II’s statistical model reveals additional excitation types obeying the same scaling (Eq. (24)), which we omit here for brevity. Summing up all these contributions we obtain a rather involved expression for ${{\mathcal{F}}}^{(k,n)}(x)$, which we omit here. The replica limit in this case is non-trivial, as the expression exhibits an explicit dependence on the replica index *n*. Taking the limit *n* → 1 − *k*, the frame potential reduces to

25 $${{\mathcal{F}}}^{(k)}(x)={{\mathcal{F}}}_{{\rm{Haar}}}^{(k)}\exp \left(xk(d-1-1/d)+x{k}^{2}\right).$$

In Fig. 3, we consider the frame potential for the first values of *k*. To access large systems’ sizes, we plot the case with *n* = 0, which corresponds to unnormalized states without Born rule, where eq. (9) can be numerically evaluated. In this case, we perform exact numerical simulations for large *N*_*A*_ by evaluating the matrices product in Eq. (9) (i.e. the so-called replica tensor network method). The plot shows that the approach to our asymptotic expression is rather slow, with corrections of order ${N}_{A}^{-1/2}$ (see inset). This fact makes a direct comparison with numerical data for the Born rule scenario (*n* = 1 − *k*) hard, as exact classical simulations of quantum systems are inherently limited to small sizes.

### The overlaps distribution

The results we derived for the frame potential can be used to obtain exact analytical expressions for the probability distribution of the (rescaled) overlaps between different states in the projected ensemble, namely ${D}_{A}| \langle \psi ({{\boldsymbol{z}}}_{B})| \psi ({\boldsymbol{z}}{\prime} )\rangle {| }^{2}$ between states on *A* conditioned on distinct measurement outcomes ${{\boldsymbol{z}}}_{B},{\boldsymbol{z}}{\prime}$ in *B*. We define

26 $$P(u)\equiv {{\mathbb{E}}}_{\psi }\left[\mathop{\sum }\limits_{\begin{array}{l}{{\boldsymbol{z}}}_{B},\\ {{{\boldsymbol{z}}}^{{\boldsymbol{{\prime} }}}}_{{\bf{B}}}\end{array}}p({{\boldsymbol{z}}}_{B})p({{{\boldsymbol{z}}}^{{\boldsymbol{{\prime} }}}}_{{\bf{B}}})\,\delta \left(u-{D}_{A}| \langle \psi ({{\boldsymbol{z}}}_{B})| \psi ({{{\boldsymbol{z}}}^{{\boldsymbol{{\prime} }}}}_{{\bf{B}}})\rangle {| }^{2}\right)\right].$$

Given that the *k*-th moment of *P*(*u*; *x*) coincide with ${{\mathcal{F}}}^{(k)},P$ is the distribution whose moments are the frame potentials. For global Haar states (*ψ* ~ Haar), the distribution takes a universal form known as the Porter-Thomas distribution, *P*_0_(*u*) = *e*^−*u*^. For the projected ensemble of RMPS, within the setup I, we instead find

27 $$P(u;x)=\frac{d-1}{d}\mathop{\sum }\limits_{j=0}^{\infty }{e}^{-\frac{u}{{z}_{j}}}{z}_{j}^{-1}{d}^{-j},$$

where ${z}_{j}=1+x\frac{d}{d-1}+xj$, and where the notation explicitly reflects the parametric dependence of the distribution on the scaling variable *x*. Clearly, in the limit of large bond dimension *χ* ≫ *D*_*A*_, we recover the Porter-Thomas distribution: $\mathop{lim}\limits_{x\to {0}^{+}}P(u;x)={P}_{0}(u)$. However, at any finite value of *x*, the distribution is non-trivial.

In the limit of large *d*, the distribution converges to a simple rescaled Porter-Thomas, as well shown in Fig. 3. In fact, the finite local physical dimension gives important fluctuations around the interface, manifested in the discrete nature of the distribution (27).

In the setup II, we instead find

28 $$P(u;x)={\int }_{-\infty }^{+\infty }\frac{dw}{\sqrt{2\pi }}{e}^{-\frac{{w}^{2}}{2}-w\sigma -\mu }\exp \left[-u{e}^{-\sigma w-\mu }\right],$$

where $\mu =x\frac{{d}^{2}-d-1}{d},\sigma =\sqrt{2x}$. This distribution arises from a suitable convolution of a Porther-Thomas distribution (first factor in Eq. (25)), and a log-normal distribution with mean *μ* and variance *σ*^2^ (second term in Eq. (25)), which, quite remarkably, has recently been shown to also appear universally in the inverse participation ratio of chaotic circuits ^49,50,67^. As specified in the previous section, large finite systems’ size corrections make the comparison with numerical data at finite *N*_*A*_ arduous, even if the scaling in *x* and the general shape are well captured at such small sizes.

### Generalization to higher-dimensional geometries

**Fig. 5:**
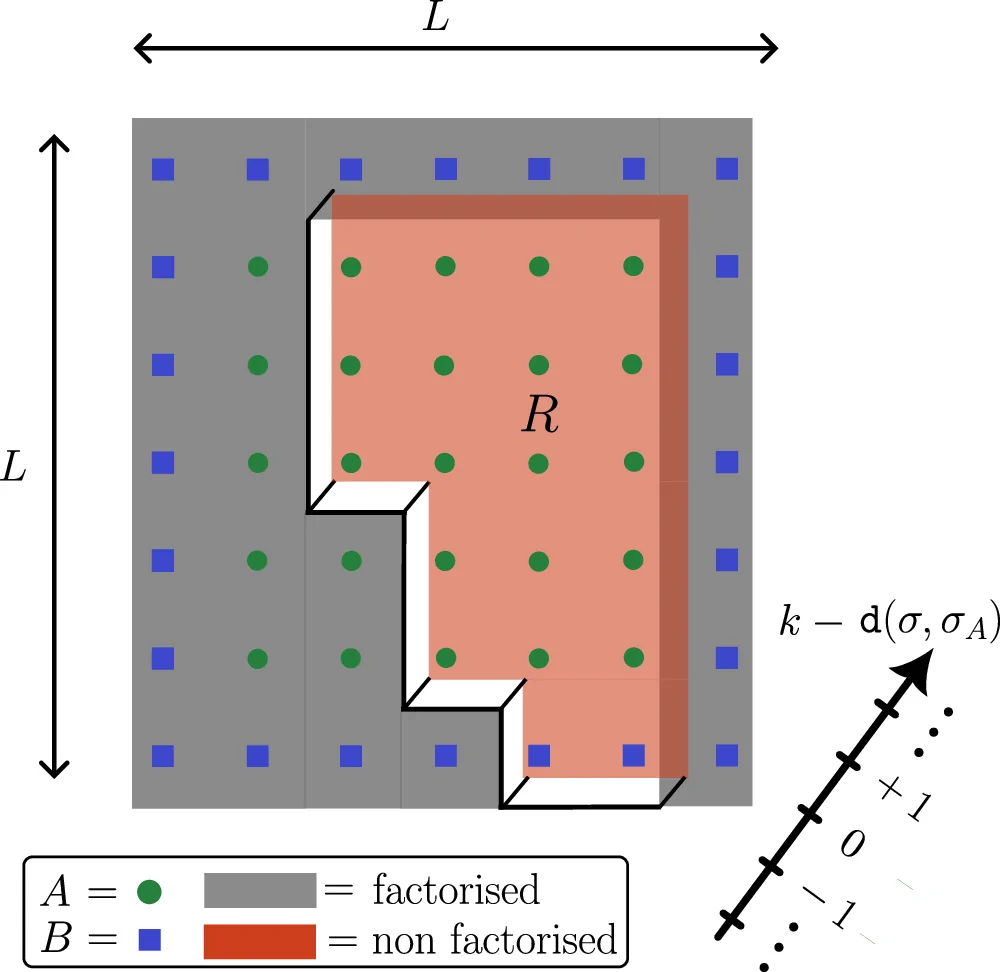
A graphical representation of domain walls excitations for the staircase RMPS (setup I), in the 2D case.

Both architectures admit a natural two-dimensional generalization on an *L* × *L* square lattice of *N* = *L*^2^ total sites. The staircase circuit architecture (setup I) can be extended to generate a 2D (random) isometric tensor network, specifically an isometric PEPS^50,69,70^. In this setting, the system is partitioned into two distinct but topologically connected regions, *A* and *B*, with measurements performed in the computational basis on one of them. Although the analytical treatment becomes more involved in two dimensions, the essential features of randomness generation via confinement remain qualitatively robust. In particular, creating a non-factorized permutation excitation over a region *R* ⊂ *A* incurs an energy cost scaling as ~*d*^∣*R*∣^/*χ*^∣∂*R*∣^. Here, *χ*^∣∂*R*∣^ is the domain wall energy cost along the boundary ∂*R* of *R*, and *d*^∣*R*∣^ reflects the energy gain from moving permutations closer to *σ*_*A*_. This structure favors extensive excitations that span large portions *R* of region *A*, while localized defects are exponentially suppressed. This suppression is overt in the scaling limit *χ*, *N*_*A*_, *L* → *∞*, with *x* ~ *D*_*A*_/*χ*^*L*^ fixed. Moreover, extended excitations may also involve parts of subsystem *B*, experiencing a similar confinement potential as in the one-dimensional case, see Fig. 5 for a sketch.

Setup *II* also admits a natural extension to two dimensions. Here, unentangled *A* blocks can be arranged on a square lattice and prepared in parallel, with subsequent gates and measurements used to “glue" them together. The resulting structure closely mirrors the one-dimensional cases: local non-factorized domain walls can be introduced by inserting a transposition on each of the four bonds surrounding a site in region *A*, at an energy cost of ${(1/{\chi }^{2})}^{4}=1/{\chi }^{8}$. This yields a defect density scaling as *x* ~ *N*_*A*_/*χ*^8^.

## Discussion

In this work, we explore the emergence of quantum randomness from local measurements on tensor network states generated by suitable quantum circuits and characterized by a finite bond dimension *χ*. Focusing on the ensembles formed by projectively measuring a portion of the system, which effectively acts as a bath to the complementary, we demonstrate that the statistical properties of the unmeasured subsystem are well described by a domain wall statistical model with confining mechanisms. We analyzed two physically motivated architectures, both compatible with near-term quantum devices. The first setup (I), uses a staircase circuit to generate a random matrix product state, which is measured on the *N*_*B*_ rightmost sites. In the second setup (II), reminiscent of recently studied glued circuits^59^, an ensemble of random MPS, forming an approximate state design with only *χ* ~ *N*^2^, is efficiently prepared by first creating unentangled blocks in parallel and then entangling them via unitaries and measurements in the *χ*^2^-dimensional auxiliary space.

In both cases, we find that confinement plays a central role in shaping the projected ensemble. In setup I, domain walls, representing interfaces between different replica configurations, are energetically pinned to the interface with the measured region. The existence of a collective confined state of domain wall can be seen as the counterpart in tensor networks of the membrane observed in random unitary circuits^39,68^. In setup II, instead, domain walls are bound into meson-like pairs that remain confined throughout the bulk. This distinction gives rise to different types of emergent randomness.

A key outcome of our analysis is the identification of a scaling regime where the projected ensemble admits exact analytical treatment. In this regime, the bond dimension *χ* scales exponentially (setup I) or, quite remarkably, polynomially (setup II) with the subsystem size, making the second proposed protocol an ideal setting for engineering approximate quantum state designs. The scaling limit yields exact analytical expressions, verified numerically whenever possible, for the frame potential and overlap distribution of the projected ensemble. Both quantities take on universal forms that remain valid far beyond infinite-depth circuits, offering directly testable predictions and implementable state design protocols for current digital quantum devices.

Interestingly, in the staircase architecture, the distribution of measurement outcomes exhibits discrete fluctuations that modulate the Porter-Thomas law, revealing a new structure in the interplay between quantum measurements and randomness. In contrast, the glued circuit provides a remarkably efficient pathway to generate approximate *k*-designs using only a polynomial bond dimension, offering a scalable route to structured quantum randomness.

Altogether, our results reveal confinement, ubiquitous in high-energy physics and in condensed matter theory, as the underlying mechanism governing the generation of randomness in quantum circuits. This work opens new avenues for understanding the structure of projected ensembles and suggests that local measurements in shallow architectures can serve as powerful tools for quantum information processing, benchmarking, and state design.

A natural question is why the measurement-based approach might be preferable to purely unitary protocols to generate quantum state designs (see, for example, Ref. ^71^). The key advantage is that measurement-based protocols allow preparation of *k*-designs *provided a single instance of the circuit’s gates*, in sharp contrast with unitary constructions, where randomness is instead induced by sampling distinct circuit’s realizations. Notice that in many practical applications, implementing distinct unitary transformations may be challenging, for instance, when the dynamics is generated by an analog Hamiltonian evolution or in a superconducting qubits platform where different gates need different calibrations. Therefore, the setups we consider have *clear physical relevance*. Concerning the specific construction of Ref. ^71^, we briefly comment on it here, as it resembles our setup II: both approaches employ two-layer brickwork circuits with gates acting on multiple qudits. In Ref. ^71^, to get a *ϵ* approximate *k* − design on *N* qubits, gates are taken to act on $2\log (N{k}^{2}/\epsilon )$. Similarly, our Eq.(25) implies *ϵ* ~ *k*^2^*N*_*A*_/*χ*^2^, so achieving a target *ϵ* requires gates acting on $2\log (\chi ) \sim \log ({N}_{A}{k}^{2}/\epsilon )$. Notice however, that in Ref. ^71^ (Corollary 1), they can then sample these unitary gates from an approximate unitary *k*-design, thus obtaining an overall circuit depth $k\,{\rm{poly}}\log (k)\log (N/\epsilon )$. In our case, instead, we need fully Haar random gates, which requires a depth ${e}^{2\log (\chi )} \sim {N}_{A}{k}^{2}/\epsilon$. While the required depth in our case scales polynomially with system size rather than logarithmically, our construction only requires a single instance of the circuit gates.

Our results also carry implications for deep thermalization rates. The scaling variables identified in Eqs. (16) and (24) directly determine the rate at which the projected ensemble converges to a *k*-design. In the circuit picture where $\chi \sim {e}^{{v}_{E}t}$, the time to reach a *k*-design scales as *t* ~ *N*_*A*_ for setup I (contiguous unmeasured subsystem) versus $t \sim \log {N}_{A}$ for setup II (non-contiguous). This suggests that non-contiguous arrangements of the unmeasured sites lead to parametrically faster deep thermalization, providing guidance for the optimal choice of measurement geometry in experiments probing thermalization dynamics.

## Methods

We aim to compute the frame potential of the projected ensemble generated by a random matrix product state (RMPS), averaged over circuit realizations. Physically, all gates in Scheme 2 and Scheme 3 are unitary and drawn from the Haar measure over the unitary group. To ease the explanation, however, we also consider a simplified ensemble in which each gate is replaced by a random matrix with i.i.d. complex Gaussian entries of zero mean and variance *ς*^2^. We refer to this as the Gauss ensemble, and denote both cases using the unified symbol ${\mathcal{E}}\in \{{\rm{Haar}},{\rm{Gauss}}\}$. As we will show, the key features of the projected ensemble remain qualitatively similar under either choice.

### Haar and Gaussian averages

Our computations require evaluating the Haar moments ${{\mathbb{E}}}_{U \sim {\rm{Haar}}(q)}[{({U}^{* }\otimes U)}^{\otimes m}]$. These can be expressed in terms of permutation operators acting on *m* copies, or replicas, of the Hilbert space^62^. We will represent them graphically as the three legs tensors  where the wavy orange line  is the permutation index *σ* ∈ *S*_*m*_, and each  leg represents the *m*-th tensor product of the physical space, thus of dimension *q*^*m*^. The central formula of Weingarten calculus reads^28,62^

$${{\mathbb{E}}}_{U \sim {\rm{Haar}}}[{({U}^{* }\otimes U)}^{\otimes m}]=\mathop{\sum }\limits_{\sigma ,\pi \in {S}_{m}}{{\rm{Wg}}}_{\sigma ,\pi }(q)|\sigma\rangle\rangle \langle\langle \pi|$$

Scheme 8where *W*(*q*) is the Weingarten matrix Wg_*σ*,*π*_(*q*) ^62^, i.e., the inverse of the Gram matrix *G*_*σ*,*π*_ = 〈〈*σ*∣*π*〉〉_*q*_ = *q*^*m*−d(*σ*, *π*)^, graphicallyScheme 9" /> We consider also matrices with i.i.d. complex Gaussian entries, with zero mean and variance *ς*^2^. The Gaussian case can always be recovered from the Haar ensemble formulas, replacing Wg_*σ*,*π*_(*q*) → *δ*_*σπ*_*ς*^2*m*^, i.e,Scheme 10leading to the Gaussian version of Weingarten formula (Wicks’ theorem)Scheme 11Note that Scheme 8 and Scheme 11 are formally the same, modulo replacing the associated matrix *W*. In fact, in the limit of large *q*, the unitary Weingarten matrix is dominated by the diagonal contribution ${{\rm{Wg}}}_{\sigma ,\pi }(q)\mathop{=}\limits^{q\to \infty }{q}^{-m}{\delta }_{\sigma ,\pi }$, implying Gauss and Haar ensembles give consistent results upon setting the variance *ς*^2^ = *q*^−1^.

### Computations of the Transfer Matrices

We present additional details on how the transfer matrices ${\widetilde{{\mathcal{T}}}}_{A}$ and ${\widetilde{{\mathcal{T}}}}_{B}$ are computed. Since the methodology is structurally similar in both setups, we detail the computation for setup I and defer the corresponding analysis for setup II. Recall each tensor is a sub-block of a matrix (gate) *U* sampled from the ensemble ${\mathcal{E}}\in \{{\rm{Haar}},{\rm{Gauss}}\}$Scheme 12By applying Eq. Scheme 8 and contracting the permutation operators acting on the *χ*-dimensional auxiliary space from two adjacent sites, we derive the following transfer matrices in the replica spaceScheme 13Scheme 14where *σ*_*A*_ is the permutation shown in Eq. (Scheme 4). The orange dot in Eq. (Scheme 13) denotes the copy tensor, forcing the three incoming permutations to be the same.

Performing the intermediate contractions, we recast Eqs. ((6), (7)).

### Confinement in the staircase circuit

We now focus on computing the frame potential for Gaussian RMPS in setup I. As we clarify at the end of this section, the same steps straightforwardly recover the Haar RMPS result, thereby complementing the analysis presented in the Results. We are interested in the scaling limit of ${{\mathcal{F}}}^{(k,n)}=({v}_{L}| {{\mathcal{T}}}_{A}^{{N}_{A}}{{\mathcal{T}}}_{A}^{{N}_{B}-1}| {v}_{R})$ for Wg_*π*,*σ*_ = *δ*_*π*,*σ*_*ς*^2*m*^ when *N*_*A*_, *χ* ≫ 1 and ${N}_{A}\ll {N}_{B}\ll {d}^{{N}_{A}}$, while the ratio

29 $$x=\frac{{d}^{{N}_{A}}}{\chi }=\frac{{D}_{A}}{\chi }\,({\rm{Gaussian}}\,{\rm{RMPS}}),$$

is kept fixed. As we will show, the results for unitary RMPS’ coincide with those of the Gaussian case, provided the scaling parameter *x* is redefined according to Eq. (16).

#### Leading order (*x* = 0)

At the leading order for *χ* → *∞*, the dominant contribution is given by the diagonal *G*_*σπ*_(*χ*) ≃ *δ*_*σπ*_*χ*^*m*^. In this limit, the transfer matrices ${{\mathcal{T}}}_{A}$ and ${{\mathcal{T}}}_{B}$ are both diagonal, while the right boundary vector for the Gaussian ensemble is an equal-weight superposition of all factorized permutations

30 $${({v}_{R})}_{\pi }\simeq {1}_{F}(\pi ){d}^{2}{\chi }^{2}{\varsigma }^{2m}.$$

Since in *A* the permutations are weighted by their distance from the permutation *σ*_*A*_, we now have to identify the subset of the factorized permutations *σ* = (*σ*_1_, *σ*_2_), *σ*_1_, *σ*_2_ ∈ *S*_*n*+*k*_, minimizing d(*σ*, *σ*_*A*_). As clarified in Ref. ^39^ (see Eq. 40 therein), these permutations always act as the identity in the *n* auxiliary replicas ${\sigma }_{1}=({{\mathbb{1}}}_{n},\alpha )$ and ${\sigma }_{2}=(\alpha {\prime} ,{{\mathbb{1}}}_{n})$, where $\alpha ,\alpha {\prime} \in {S}_{k}$. Moreover the two permutations *α* and $\alpha {\prime}$ are related by an inversion, $({{\mathbb{1}}}_{k},\alpha {\prime} )={i}_{2k}({\alpha }^{-1},{{\mathbb{1}}}_{k}){i}_{2k}$. Thus, there are precisely *k*! factorized permutations minimizing the distance from the permutation *σ*_*A*_. It can also be proven that for all of them d(*σ*, *σ*_*A*_) = *k*. Collecting these remarks, we have at leading order in *χ*

31 $${{\mathcal{F}}}^{(k,n,0)}:=\mathop{{\mathrm{lim}}}\limits_{\chi \to \infty }{{\mathcal{F}}}^{(k,n)}=k!\frac{{\chi }^{2}{d}^{(m-k){N}_{A}+2{N}_{B}}}{{\chi }^{m(1-N)}}{\varsigma }^{2mN}$$

Substituting *ς*^2^ = (*dχ*)^−1^ into Eq. (31) yields the frame potential for the Haar ensemble of RMPS. At leading order, this reproduces the Haar frame potential ${{\mathcal{F}}}^{(k,1-k,0)}=k!\,{D}_{A}^{-k}={{\mathcal{F}}}_{{\rm{Haar}}}^{(k)}$.

#### Domain wall picture in the scaling limit (finite *x*)

We now consider the full calculation of ${{\mathcal{F}}}^{(k)}$, in the scaling limit Eq. (29), for a fixed finite value of *x*. The discussion here for the Gaussian case is a detailed version of the one in Sec. for the unitary Haar case. Let us expand the Gram matrix in powers of 1/*χ*

32 $${G}_{\sigma \pi }(\chi )={\chi }^{m}\left({\delta }_{\sigma \pi }+\mathop{\sum }\limits_{\alpha \ge 1}{\chi }^{-\alpha }{{\mathsf{A}}}_{\sigma \pi }^{(\alpha )}\right),\,{{\mathsf{A}}}_{\sigma \pi }^{(\alpha )}={\delta }_{{\mathtt{d}}(\sigma ,\pi ),\alpha }\,.$$

which represents the Gaussian analog of Eq. (11). Note that ${{\mathsf{A}}}_{\sigma \pi }^{(1)}={{\mathsf{A}}}_{\sigma \pi }$ is the adjacency matrix of permutations introduced in the main text, indicating whether *σ* and *π* differ by a single transposition. As discussed in the main text, expanding ${{\mathcal{F}}}^{(k,n)}$ at first order in 1/*χ* amounts to replacing one identity with ${{\mathsf{A}}}^{(1)}$ (compare with Eq. (15)). This corresponds to a domain wall configuration *σ*_1_ ⋯ *σ*_1_ *σ*_0_ ⋯ *σ*_0_ where the permutations *σ*_1_ and *σ*_0_ differ by a single transposition. Consistent with Eq. (30), the right boundary forces *σ*_0_ to be a factorized permutation. The domain wall carries a cost of *χ*^−1^, which must be offset by a sufficient number of factors of *d* obtained in the region *A* to survive in the scaling limit (29). This compensation occurs if the distance to *μ*_*A*_ decreases from d(*σ*_0_, *μ*_*A*_) = *k* to d(*σ*_1_, *μ*_*A*_) = *k* − 1. This condition necessarily implies that *σ*_1_ is non-factorized. Writing *σ*_0_ = *τ*_1_,…,*τ*_*k*_*σ*_*A*_, where {*τ*_*i*_} is a set of commuting transpositions, we see that there are *k* choices for *σ*_1_, removing any of the *τ*_*i*_. At first order in 1/*χ*, this produces the same result as in Eq. (17).

Let us discuss higher order in *O*(*χ*^−*α*^) and thus of *O*(*x*^*α*^) in the scaling limit. One can have insertions of *α* simple domain walls associated with ${{\mathsf{A}}}^{(1)}$ at distinct bonds *ℓ*_*α*_ < *ℓ*_*α*−1_ < … < *ℓ*_1_, each associated with the removal of a transposition *τ* ∈ {*τ*_*i*_} defining *σ*_0_. There are *k*!/(*k* − *α*)! ways to choose this ordered subset of transpositions out of the *k* transpositions {*τ*_*i*_}. However, it is also possible to have insertions of composed domain walls associated with the higher-order matrices ${{\mathsf{A}}}^{(p)}$. To see their effect explicitly, let us analyze in detail the case *α* = 2. When the two domain walls (transpositions) occur at distinct positions *ℓ*_1_ < *ℓ*_2_, there are $\frac{k!}{(k-\alpha )!}=k(k-1)$ possible ordered arrangements for the transpositions *τ*_1_, …, *τ*_*α*_. In fact, the order matters, as for instance placing *τ*_1_ at *ℓ*_1_ and *τ*_2_ at *ℓ*_2_ results in a different configuration from placing *τ*_2_ at *ℓ*_1_ and *τ*_1_ at *ℓ*_2_. In contrast, the insertion of a single matrix ${{\mathsf{A}}}_{{\tau }_{1}{\tau }_{2}{\sigma }_{A},{\sigma }_{A}}^{(2)}$ can be identified with the coinciding positions *ℓ*_1_ = *ℓ*_2_. Overall, we obtain

33 $$k(k-1)\mathop{\sum }\limits_{{\ell }_{1} < {\ell }_{2}}(\ldots )+\frac{k(k-1)}{2}\mathop{\sum }\limits_{{\ell }_{1}={\ell }_{2}}(\ldots )=\left(\begin{array}{c}k\\ 2\end{array}\right)\mathop{\sum }\limits_{{\ell }_{1},{\ell }_{2}}(\ldots ),$$

where, on the right-hand side of the equality, we have extended the summation to all values of *ℓ*_1_, *ℓ*_2_ without constraints, thereby factorizing the combinatorial factor $$\left(\begin{array}{c}k\\ \alpha \end{array}\right)$$ with *α* = 2. It is easy to verify from the simple form of the matrices ${{\mathsf{A}}}^{(\alpha )}$(32) that this discussion generalizes at higher orders, so that accounting for composite domain walls is done easily by extending the sum over *ℓ*_1_, …, *ℓ*_*α*_ to be unconstrained, thus including coinciding points (see also the analogous discussion in Sec. IID.2 in ref. ^66^). With these considerations in mind, we arrive at the following expansion

34 $${{\mathcal{F}}}^{(k)}(x)={{\mathcal{F}}}_{{\rm{Haar}}}^{(k)}\mathop{\sum }\limits_{\alpha =0}^{k}\left(\begin{array}{c}k\\ \alpha \end{array}\right)\,{x}^{\alpha }{f}_{\alpha },$$

where the sum runs over the index *α* labeling the number of domain walls. By construction, *f*_0_ = 1, as discussed in the previous section and in general

35 $${f}_{\alpha }=\mathop{\sum }\limits_{{\ell }_{1},\ldots ,{\ell }_{\alpha }=-\infty }^{\infty }{d}^{-V({\ell }_{1},\ldots ,{\ell }_{\alpha })}$$

where *V*(*ℓ*_1_, …, *ℓ*_*α*_) = *V*_*A*_(*ℓ*_1_, …, *ℓ*_*α*_) + *V*_*B*_(*ℓ*_1_,…,*ℓ*_*α*_) are the effective confining potential in Eq. (20). To evaluate *f*_*α*_ as a function of *d*, let us split the sums over positive (in *B*) and negative (in *A*) *ℓ*’s, defining the coefficients *a*_*α*_ and *b*_*α*_, given by *a*_0_ = *b*_0_ = 1 and

36 $$\begin{array}{l}{a}_{\alpha }\,:=\,\mathop{\sum }\limits_{{\ell }_{1},\ldots ,{\ell }_{\alpha }=0}^{-\infty }{d}^{-{V}_{A}({\ell }_{1},\ldots ,{\ell }_{\alpha })}={\left(\frac{d}{d-1}\right)}^{\alpha },\\ {b}_{\alpha }\,:=\,\mathop{\sum }\limits_{{\ell }_{1},\ldots ,{\ell }_{\alpha }=1}^{+\infty }{d}^{-{V}_{B}({\ell }_{1},\ldots ,{\ell }_{\alpha })}=\frac{d-1}{d}{\sum }_{j=1}^{+\infty }{d}^{-j}{j}^{\alpha }.\end{array}$$

for *α*≥1. In the above expressions, we substituted Eq. (20) and performed the explicit computation. Using Eq. (36), we can decompose *f*_*α*_ into a sum over *β*, the number of domain walls in *B*

37 $${f}_{\alpha }=\mathop{\sum }\limits_{\beta =0}^{\alpha }\left(\begin{array}{c}\alpha \\ \beta \end{array}\right){a}_{\alpha -\beta }{b}_{\beta }={\left(\frac{d}{d-1}\right)}^{\alpha -1}\left(1+\mathop{\sum }\limits_{j=1}^{+\infty }{d}^{-j}{\left(1+j\frac{d-1}{d}\right)}^{\alpha }\right)\,,$$

where *β* is the number of particles in *B*. Plugging this expression in Eq. (34), we arrive at

38 $${{\mathcal{F}}}^{(k)}(x)={{\mathcal{F}}}_{{\rm{Haar}}}^{(k)}\frac{d-1}{d}\mathop{\sum }\limits_{j=0}^{+\infty }{d}^{-j}{\left(1+x\frac{d}{d-1}+xj\right)}^{k}.$$

Recall that ${{\mathcal{F}}}^{(k)}$ is, up to rescaling, the *k*-th moment of the random variable *u*, distributed according to Eq. (26). To recover the distribution, it is useful to identify the moments as obtained from the decomposition *u* = *u*_0_*z* in terms of two independent random variables: a Porter-Thomas distributed variable *u*_0_ with probability ${p}_{{u}_{0}}({u}_{0})={e}^{-{u}_{0}}$ and moments ${\mathbb{E}}[{u}_{0}^{k}]={D}_{A}^{k}{{\mathcal{F}}}_{{\rm{Haar}}}^{(k)}=k!$, and a discrete random variable *z*, with distribution ${p}_{z}(z)=(d-1){\sum }_{j=0}^{+\infty }{d}^{-j-1}\delta (z-{z}_{j})$ and moments ${\mathbb{E}}[{z}^{k}]=(d-1){\sum }_{j=0}^{+\infty }{d}^{-j+1}{({z}_{j})}^{k}$ where ${z}_{j}=1+x\frac{d}{d-1}+xj$. As a result, the distribution of *u* is the multiplicative convolution of ${p}_{{u}_{0}}$ and *p*_*z*_, namely

39 $$P(u;x)={\int }_{-\infty }^{+\infty }\frac{dt}{| t| }{p}_{{u}_{0}}(t){p}_{z}\left(\frac{u}{t}\right)=\frac{d-1}{d}\mathop{\sum }\limits_{j=0}^{\infty }{e}^{-\frac{u}{{z}_{j}}}{z}_{j}^{-1}{d}^{-j}.$$

Note that *P*(*u*; *x*) is properly normalized, since ${\int }_{0}^{\infty }du\,{e}^{-\frac{u}{{z}_{j}}}{z}_{j}^{-1}=1$ and $\frac{d-1}{d}{\sum }_{j=0}^{\infty }{d}^{-j}=1$, and depends parametrically on the scaling variable *x*. In particular, for *x* → 0 we recover the Porter-Thomas distribution ${{\mathrm{lim}}}_{x\to {0}^{+}}P(u;x)={e}^{-u}$ since ${{\mathrm{lim}}}_{x\to {0}^{+}}{z}_{j}=1$ for all *j*.

#### Unitary case

We can now extend our results to the ensemble of Haar unitary RMPS. This extension requires to handle the non-trivial expression of the Weingarten matrix in Eqs. Scheme 13. Crucially, the product of transfer matrices ${{\mathcal{T}}}_{A},{{\mathcal{T}}}_{B}$ depends only on *T*(*χ*, *d*) = Wg(*dχ*)*G*(*χ*). The Weingarten matrix acts as a dressing of the ferromagnetic interaction between adjacent sites in the Gaussian case encoded in *G*(*χ*). Remarkably, it is still possible to obtain a closed expression in the scaling limit. To see this, we note that in the scaling limit (16), as we discussed in, the relevant configurations in the scaling limit at the order *O*(*x*^*α*^) contain *α* domain walls at positions *ℓ*_*α*_, *ℓ*_*α*−1_, …, *ℓ*_1_; thus, they are made of *α* + 1 domains, *σ*_*α*_, *σ*_*α*−1_, …, *σ*_1_, *σ*_0_ = *σ* with $\sigma \in {S}_{m}^{{\rm{fact}}}$ and d(*σ*_*a*_, *σ*_*A*_) = *k* − *a*. As discussed in the previous section, higher orders in the *χ* → *∞* expansion of *T*(*χ*, *d*) can be associated with cases where *ℓ*_*i*_ = *ℓ*_*i*+1_ = … = *ℓ*_*i*+*β*−1_. This corresponds to the composite domain wall between *σ*_*i*+*β*−1_ and ${\sigma }_{i-1}={\tau }_{{m}_{1}}\ldots {\tau }_{{m}_{\beta }}{\sigma }_{i+\beta -1}$, so that d(*σ*_*i*−1_, *σ*_*i*+*β*−1_) = *β*. The associated cost is then given by ${T}_{{\sigma }_{i+\beta -1},{\sigma }_{i-1}}(\chi ,d)={\chi }^{-\beta }{d}^{-m}{{\mathsf{T}}}_{{\sigma }_{i+\beta -1},{\sigma }_{i-1}}^{(\beta )}+O({\chi }^{-\beta -1})$. This term can be explicitly computed from the known expansion of Weingarten functions. Specifically, setting *σ* = *σ*_*i*+*β*−1_ and ${\sigma }^{{\prime} }={\sigma }_{i-1}={\tau }_{{m}_{1}}\ldots {\tau }_{{m}_{\beta }}\sigma$, we have

40 $${T}_{\sigma ,{\sigma }^{{\prime} }}(\chi ,d)=\mathop{\sum }\limits_{\pi \in {S}_{m}}{[\mathrm{Wg}(d\chi )]}_{{\sigma }^{{\prime} }\pi }{[G(\chi )]}_{\pi \sigma }.$$

At the leading order *O*(*χ*^−*β*^), the sum over *π* is dominated by the cases when the intermediate permutation $\pi ={\tau }_{{g}_{1}}\ldots {\tau }_{{g}_{b}}\sigma$, where ${\{{\tau }_{{g}_{i}}\}}_{i=1}^{b}$ are a subset of size *b* of ${\{{\tau }_{{m}_{i}}\}}_{i=1}^{\beta }$. This implies

41 $${[\mathrm{Wg}(d\chi )]}_{{\sigma }^{{\prime} }\pi }={(\chi d)}^{-m}\left(\frac{{(-1)}^{\beta -b}}{{d}^{\beta -b}{\chi }^{\beta -b}}+O({(d\chi )}^{\beta -b+1})\right)$$

Finally, since *d*(*σ*, *π*) = *b*, we have *G*_*πσ*_(*χ*) = *χ*^*m*−*b*^. Summing over the possible choices of the *b* transpositions among the *β* ones, we obtain

42 $${{\mathsf{T}}}_{{\sigma }^{{\prime} }\sigma }^{(\beta )}=\mathop{\sum }\limits_{b=0}^{\beta }\left(\begin{array}{c}\beta \\ b\end{array}\right)\frac{{(-1)}^{b}}{{d}^{b}}={\left(\frac{d-1}{d}\right)}^{\beta }$$

which shows that (*d* − 1)/*d* effectively renormalizes the interaction strength. Overall, this amounts only to redefining the scaling variable *x* ↦ *x*(*d* − 1)/*d*, consistent with Eq. (16) in the Main Text.

### Derivation of the frame potential for setup II

We now present the detailed computation of the generalized frame potential ${{\mathcal{F}}}^{(k,n)}$ for setup II. We begin with the Gaussian ensemble for analytical tractability and then recover the Haar result. The relevant scaling limit is *x* = *N*_*A*_/*χ*^2^.

*Leading order* (*x* = 0). At leading order for *χ* → *∞*, the dominant contributions come from factorized permutations minimizing the distance from *σ*_*A*_. Setting the Gaussian variance to ${\varsigma }_{A}^{2}={(d{\chi }^{2})}^{-1}$ for gates on *A* sites and ${\varsigma }_{B}^{2}={({\chi }^{2})}^{-1}$ for *B* sites, one obtains ${{\mathcal{F}}}^{(k,n,0)}=k!\,{d}^{-k{N}_{A}}{\chi }^{(4-2m)({N}_{A}+1)}$. In the replica limit *m* → 2, this recovers the Haar value ${{\mathcal{F}}}^{(k,1-k,0)}=k!\,{D}_{A}^{-k}={{\mathcal{F}}}_{{\rm{Haar}}}^{(k)}$.

*Domain wall picture* (*finitex*). We classify all excitation types above the *k*! vacuum states:

**A1** These excitations are localized on a single *A* site. A Gram matrix insertion induces a transposition reducing d(*σ*, *σ*_*A*_) from *k* to *k* − 1, at cost 1/*χ*, with multiplicity *k*. To avoid a penalty of order *χ*^2^ on the subsequent *B* site (since the resulting permutation is non-factorized), the excitation must be immediately removed, contributing an additional 1/*χ*. Total contribution: *x* ⋅ *kd*.

**F1** These excitations transition one vacuum state *σ* to another factorized permutation at distance *k* + 1 from *σ*_*A*_, persisting over *ℓ* sites with cost 1/*d* per *A* site. Summing over all lengths *ℓ* and starting positions yields a factor (*d* + 3)/(*d* − 1). The permutation multiplicity is *m*(*m*/2 − 1), since one can choose among $\frac{m}{2}(\frac{m}{2}-1)$ factorization-preserving transpositions and return via the same or inverse transposition. Contribution: $x\cdot \frac{d+3}{d-1}\,m(\frac{m}{2}-1)$.

**F2** These arise from contact between two distinct vacuum states $\sigma {\prime} ,\sigma$ at distance ${\mathtt{d}}(\sigma {\prime} ,\sigma )=2$. The Gram matrix produces a double jump (two transpositions) transitioning between vacuum states at cost 1/*χ*^2^. Multiplicity as in **F1** but with *m* → *k* (all permutations are factorized). Contribution: *x* ⋅ *k*(*k* − 1).

**A2** These involve a single *A* site, bringing a vacuum state to a permutation at distance *k* + 1 from *σ*_*A*_ at cost 1/*d*. The multiplicity is $m(m-1)/2-k-\frac{m}{2}(\frac{m}{2}-1)$, accounting for all transpositions minus those already counted in **A1** and **F1**. Contribution: $x\cdot \frac{1}{d}\left[\frac{m(m-1)}{2}-k-\frac{m}{2}(\frac{m}{2}-1)\right]$.

Since excitations are dilute in the scaling limit and can be created independently, we exponentiate the first-order correction to obtain the Gaussian result:

43 $${{\mathcal{F}}}^{(k,n)}={{\mathcal{F}}}^{(k,n,0)}\exp \left[x\left(kd+\frac{1}{d}\left(\frac{m(m-1)}{2}-k-\frac{m}{2}\left(\frac{m}{2}-1\right)\right)+m\left(\frac{m}{2}-1\right)\frac{d+3}{d-1}+k(k-1)\right)\right].$$

*Unitary case*. Two additional contributions arise from the Weingarten expansion:

**U1** The Weingarten constant ${c}^{(W)}(q)={q}^{-m}(1-\frac{m(m-1)}{2q}+\cdots )$ at *A* sites with *q* = *dχ*^2^ contributes $-x\,\frac{m(m-1)}{2d}$.

**U2** At *B* sites, the Weingarten matrix Wg(*χ*^2^) can insert a transposition letting one vacuum state transition to another factorized permutation at distance 1, with multiplicity $\frac{m}{2}(\frac{m}{2}-1)$. Contribution: $-x\,\frac{m}{2}(\frac{m}{2}-1)$.

Including these, the full unitary result reads

44 $$\begin{array}{ll}{{\mathcal{F}}}^{(k,n)}={{\mathcal{F}}}^{(k,n,0)}\exp \left[x\left(kd+\frac{1}{d}\left(\frac{m(m-1)}{2}-k-\frac{m}{2}\left(\frac{m}{2}-1\right)\right)\right.\right.\\ \quad\quad\quad\quad\quad\left.\left.+m\left(\frac{m}{2}-1\right)\frac{d+3}{d-1}+k(k-1)-\frac{m(m-1)}{2d}-\frac{m}{2}\left(\frac{m}{2}-1\right)\right)\right].\end{array}$$

Taking the replica limit *n* → 1 − *k* (*m* → 2), we recover Eq. (25):

45 $${{\mathcal{F}}}^{(k,1-k)}={{\mathcal{F}}}_{{\rm{Haar}}}^{(k)}\exp \left[x\left({k}^{2}+k\frac{{d}^{2}-d-1}{d}\right)\right].$$

## Data Availability

The data for the results presented in this work will be publicly available at publication.
